# Metabolomic analysis reveals potential role of immunometabolism dysregulation in recurrent pregnancy loss

**DOI:** 10.3389/fendo.2024.1476774

**Published:** 2024-10-09

**Authors:** Xiaofeng Ye, Chong Ma, Wenqi Guo, Yan Guo, Dong-dong Li, Sihang Zhou, Qingyu Hu, Yanjun Hong, Zhiyong Xie, Liping Wang

**Affiliations:** ^1^ Reproductive Medicine Centre, The First Affiliated Hospital of Shenzhen University, Shenzhen Second People's Hospital, Guangdong Key Laboratory for Biomedical Measurements and Ultrasound Imaging, National-Regional Key Technology Engineering Laboratory for Medical Ultrasound, School of Biomedical Engineering, Shenzhen University Medical School, Shenzhen, China; ^2^ School of Pharmaceutical Sciences (Shenzhen), Sun Yat-sen University, Shenzhen, China; ^3^ Department of Gynecology and Obstetrics, Huiyang District Maternal and Child Health Care Hospital, Huizhou, China; ^4^ Department of Gynecology and Obstetrics, Graduate College of Guangxi University of Traditional Chinese Medicine, Nanning, China; ^5^ Reproductive Medicine Centre, The First Affiliated Hospital of Shenzhen University, Shenzhen Second People's Hospital, Shenzhen, China; ^6^ Health Science Center, Shenzhen University, Shenzhen, China

**Keywords:** recurrent pregnancy loss, immunometabolism, metabolomics, amino acid metabolism, immune cytokines, biomarkers, mechanisms

## Abstract

**Background:**

Recurrent pregnancy loss (RPL) affects women's reproductive health seriously, with immune dysfunction playing a key role in its cause, yet the exact mechanisms remain elusive. We aim to investigate potential mechanisms and identify biomarkers linked to RPL.

**Methods:**

Immune cytokine testing and metabolomic profiling were conducted on the serum of 34 RPL patients and 30 healthy individuals. The metabolic pathways of the differential metabolites were analyzed, and specific metabolites were validated through targeted profiling. Potential biomarkers were identified, and the relationships between immune cytokines and differential metabolites were explored.

**Results:**

In the RPL group, serum interleukin-6 and interleukin-10 levels were significantly higher, while interleukin-2 and interferon-γ were significantly lower. A total of 296 differential metabolites were detected by untargeted metabolomic profiling between the RPL and control groups, with most linked to amino acid metabolism. Targeted metabolomic profiling of amino acid metabolism revealed upregulation of indole-3-acetic acid, tyrosine, glycine, isoleucine, tryptophan, lysine, aspartic acid, arginine, leucine, threonine, glutamic acid, cystine, and phenylpyruvic acid (PPA) in the RPL group. Moreover, PPA and 5-hydroxy-L-tryptophan showed great potential in predicting RPL in a diagnostic model. Cystine and tyrosine were associated with immune cytokines in correlation analysis.

**Conclusion:**

The study highlights the role of amino acid metabolism in RPL pathogenesis, suggesting that PPA and 5-HTP may be potential predictive indicators, while cystine and tyrosine may potentially regulate immune responses related to RPL. Further investigation into the molecular mechanisms underlying these findings could potentially result in the creation of novel diagnostic and therapeutic approaches for RPL.

## 1 Introduction

Recurrent pregnancy loss (RPL) is a serious and complex complication of pregnancy. The definition of RPL is currently not standardized globally. Per the recommendations of the European Society of Human Reproduction and Embryology (ESHRE), RPL is defined as the occurrence of two or more clinically recognized pregnancies ending before 24 weeks of gestation, and excluding ectopic and molar pregnancies (1). RPL affects around 2.5% of women attempting to conceive (2). Research indicates that women who have experienced pregnancy loss have a heightened risk, up to 75%, of subsequent losses, significantly impacting their physical and mental health (3). The causes of RPL include genetics, immune dysfunction, endocrine issues, infections, coagulation abnormalities, and anatomical irregularities. Reports suggest that 50% of RPL cases are linked to immune dysfunction, particularly involving type 1 helper T cell (Th1) and type 2 helper T cell (Th2) dysregulation (4, 5). Currently, the frequency of pregnancy losses is the diagnostic criterion for RPL, with each loss inflicting significant physical and psychological harm to women. By the time they meet the diagnostic criteria for RPL in their country, many women have already given up on the possibility of a successful pregnancy. Given the significant impact of RPL, it is imperative to find biomarkers to identify individuals at risk of RPL before the condition manifests, and fully investigate its mechanisms to develop effective treatment approaches.

Metabolomics is a promising technique for analyzing all low molecular weight metabolites in samples qualitatively and quantitatively. Therefore, metabolomics can be utilized to uncover mechanisms and identify potential biomarkers (6). Currently, there is limited research on the metabolomics of individuals with RPL. Li’s study found elevated lactic acid and reduced 5-methoxytryptamine in the first trimester of RPL patients compared to controls (7). A survey of RPL in women with antiphospholipid syndrome identified five biomarkers associated with various metabolic pathways, including amino acid, purine, and tyrosine metabolism (8). Additionally, in Iranian women with RPL, there were notable variances in hypotaurine and taurine metabolism and tyrosine, phenylalanine, and tryptophan biosynthesis (9). Another study highlighted oxidative stress as a crucial pathway in the development of RPL (10). Analysis of women with RPL during the implantation window suggests that specific metabolites may affect endometrial receptivity by influencing reduced inflammatory responses and vascular dysregulation (11). However, previous studies based on untargeted metabolomic analyses of nonpregnancy RPL patients have limitations in their relative quantification results. Absolute quantification of specific pathways in clinical cohort samples is crucial for identifying clinical diagnostic and therapeutic biomarkers in the future.

In our research, immune cytokines were assessed to monitor the immune status of Th1 and Th2 in RPL, and distinct metabolomics were identified through untargeted and targeted metabolomic profiling in nonpregnancy RPL and healthy participants. Additionally, we conducted subgroup analyses of serum metabolites in primary and secondary RPL, as well as in subgroups based on the number of pregnancy losses. Functional enrichment analysis of differential metabolites was conducted. Furthermore, diagnostic models were developed in differential metabolites, and an association investigation was conducted between immune cytokines and metabolites. Our study aimed to pinpoint unique metabolites that may act as innovative markers for predicting RPL and investigate new mechanisms and pathways implicated in the development of RPL.

## 2 Materials and methods

### 2.1 Study participants

This study received approval from the Shenzhen Second People’s Hospital Institutional Review Board (2024-194-01PJ). Each participant provided written informed consent. According to the ESHRE guideline (2022), patients meeting the following three conditions were defined as RPL: (a) loss of two or more either urine or serum β-hCG confirmed pregnancies, encompassing treated pregnancies of unknown location or biochemical pregnancy loss; (b) pregnancy loss occurring from conception up to 24 weeks of gestation; (c) ectopic, molar pregnancies, and implantation failure were excluded (1). In addition, participants’ ages ranged from 20 to 40 years. Women who had either experienced successful full-term pregnancies or were healthy individuals with no history of pregnancy loss were included as controls. Additionally, those suffering from (a) pregnancy status; (b) chromosome karyotype abnormalities; (c) anatomical cacogenesis of the uterus, such as uterine malformation, submucosal myoma, endometrial polyps (> 5mm), and intrauterine adhesions; or those with an intrauterine device; (d) RPL caused by male factors; (e) women with polycystic ovary syndrome, hyperprolactinemia, autoimmune diseases, severe heart, liver, or kidney dysfunction, or a history of cancer were excluded from the study. Finally, our study involved 64 participants, with 30 in the control group and 34 in the RPL group recruited from the Reproductive Medicine Centre, The First Affiliated Hospital of Shenzhen University, Shenzhen Second People’s Hospital.

### 2.2 Clinical data collection and sample collection

Participants were thoroughly interviewed, and their pregnancy history was documented. Most of the participants underwent blood tests, including thyroid-stimulating hormone (TSH) and anti-Müllerian hormone (AMH). Morning fasting blood samples (a minimum of 3 mL per person) were collected from participants using gel separator tubes. The samples were then centrifuged at 1500 × g for 15 minutes at room temperature to separate the serum, which was subsequently preserved at -80°C until required for analysis.

### 2.3 Cytokines assay

Serum levels of interleukin-2 (IL-2), interferon-γ (IFN-γ), tumor necrosis factor-α (TNF-α), interleukin-6 (IL-6), interleukin-4 (IL-4), and interleukin-10 (IL-10) were measured via enzyme-linked immunosorbent assay (ELISA) kits (QUANZHOU RUIXIN BIOTECHNOLOGY CO. LTD., Quanzhou, China) following the producer’s protocols. The IL-6 and IL-4 ELISA tests have a detection range of 1.5 to 48 pg/mL, while the IL-2, IL-10, and IFN-γ ELISA tests have a range of 25 to 800 pg/mL. The TNF-α ELISA test covers a range of 2.5 to 80 pg/mL. The inter-assay and intra-assay variations of cytokines are < 15% and < 10%, separately. The recognition limit for the IL-4, IL-6, and TNF-α ELISA kits is 0.1 pg/mL, whereas for the IL-2, IL-10, and IFN-γ ELISA kits, it is 1 pg/mL. All standard curves had R² values > 0.99.

### 2.4 Untargeted metabolomic profiling

Untargeted metabolomics analysis was executed using UHPLC-QTOF-MS/MS, according to methods described previously (12). In brief, 50 μL of serum sample was mixed with 450 μL of pre-cooled HPLC-grade acetonitrile, followed by vortexing for 2 minutes, left to stand for 20 minutes at 4°C, and centrifuged at 13523 × g for 15 minutes at 4°C. The supernatant was collected for metabolomic profiling. Additionally, quality control (QC) sample was prepared by pooling 10 μL from each serum sample via the same methods. All samples were analyzed in a random order.

The chromatographic examination separation was performed via a Waters AC-QUITY UPLC system with an ACQUITY HSS T3 column (100 mm × 2.1 mm, 1.8 μm). The MS data was acquired using a SYNAPT G2-Si HDMS Q-TOF-MS instrument (Waters Corporation, Milford, MA, United States) in both negative and positive electrospray ionization modes. Detailed parameter settings can be found in a previously published article (12).

The data was processed in Progenesis QI V2.0 software to remove background noise and align peaks. Peaks with above 50% missing values were removed, and padding with zeros was applied using the k-nearest Neighbor method. Normalization was carried out in the MetFlow Platform using the QC SVR (MetNormalizer). Multivariate data analysis, including principal component analysis (PCA) and partial least squares discriminant analysis (PLS-DA), was conducted using the OmicStudio tools (https://www.omicstudio.cn/tool) (13). The reliability of the PLS-DA models was assessed through permutation tests. Distinctive metabolites between groups were selected based on the following criteria: fold change (FC) > 2.0, adjusted p-values using the T-test method < 0.05, and variable importance in the projection (VIP) > 1 in PLS-DA. Each peak was identified by matching the accurate mass and MS/MS fragments with benchmark data from the human metabolome database. Pathway analysis of the differential metabolites was conducted utilizing MetaboAnalyst 6.0 and the Kyoto Encyclopedia of Genes and Genomes (KEGG) database (14).

### 2.5 Targeted metabolomic profiling

Targeted metabolomic profiling of 46 serum amino acids was performed as previously reported, including phenlpyruvic acid (PPA), cystine, arginine, threonine, aspartic acid, lysine, indole-3-acetic acid (IAA), glycine, tyrosine, glutamic acid, leucine, tryptophan, isoleucine, indole-3-carboxylic acid, urocaninic acid, 4-hydroxybenzoic acid, phenylalanine, glntamine, histidine, ornithine, proline, alanine, methionine, valine, serine, phenyllactic acid, indole-3-lactic acid, benzoic acid, indole-3-propionic acid, histamine, phenylacetic acid, 3-hydroxybenzoic acid, tyrosol, imidazolepropionate, indole-3-carboxaldehyde, hydroxycinamic acid, 4-hydroxyphenylacetic acid, tryptamine, tryptophol, 4-hydroxycinamic acid, 4-hydroxyphenlpyruvic acid, indole-3-acetamide, hippuric acid, indole-3-acrylic acid, indole-3-pyruvic acid, indole-3-butyric acid (15–17). Briefly, 10 μL of internal standard (paminosalicylic acid) was added to 50 μL of serum sample. After incubation for 5 minutes on ice, 450 μL of HPLC-grade pre-cooled acetonitrile was added. The mixture was vortexed for 2 minutes, then left to stand for 20 minutes at 4°C, and centrifuged for 15 minutes at 13523 × g at 4°C. The supernatant was collected for UHPLC-QQQ-MS/MS analysis. All standard curves had R² values > 0.99. Distinctive metabolites between the groups were selected by p < 0.05 and |log2(FC)| > 0.25.

### 2.6 Statistical analysis

The baseline characteristics of the subjects were examined via the Statistical Product and Service Solutions software (SPSS V24.0, Chicago, IL, United States). Variables were analyzed by student’s t-test. Receiver operating characteristic (ROC) analysis, random forest model, and Pearson’s correlation coefficient were carried out by the OmicStudio tools at https://www.omicstudio.cn/tool (13). GraphPad Prism was utilized for data visualization. The results were presented as mean ± standard deviation, and a two-tailed p-value < 0.05 was considered statistically significant.

## 3 Results

### 3.1 Clinical characteristics and immune cytokines of the participants

**Figure 1:**
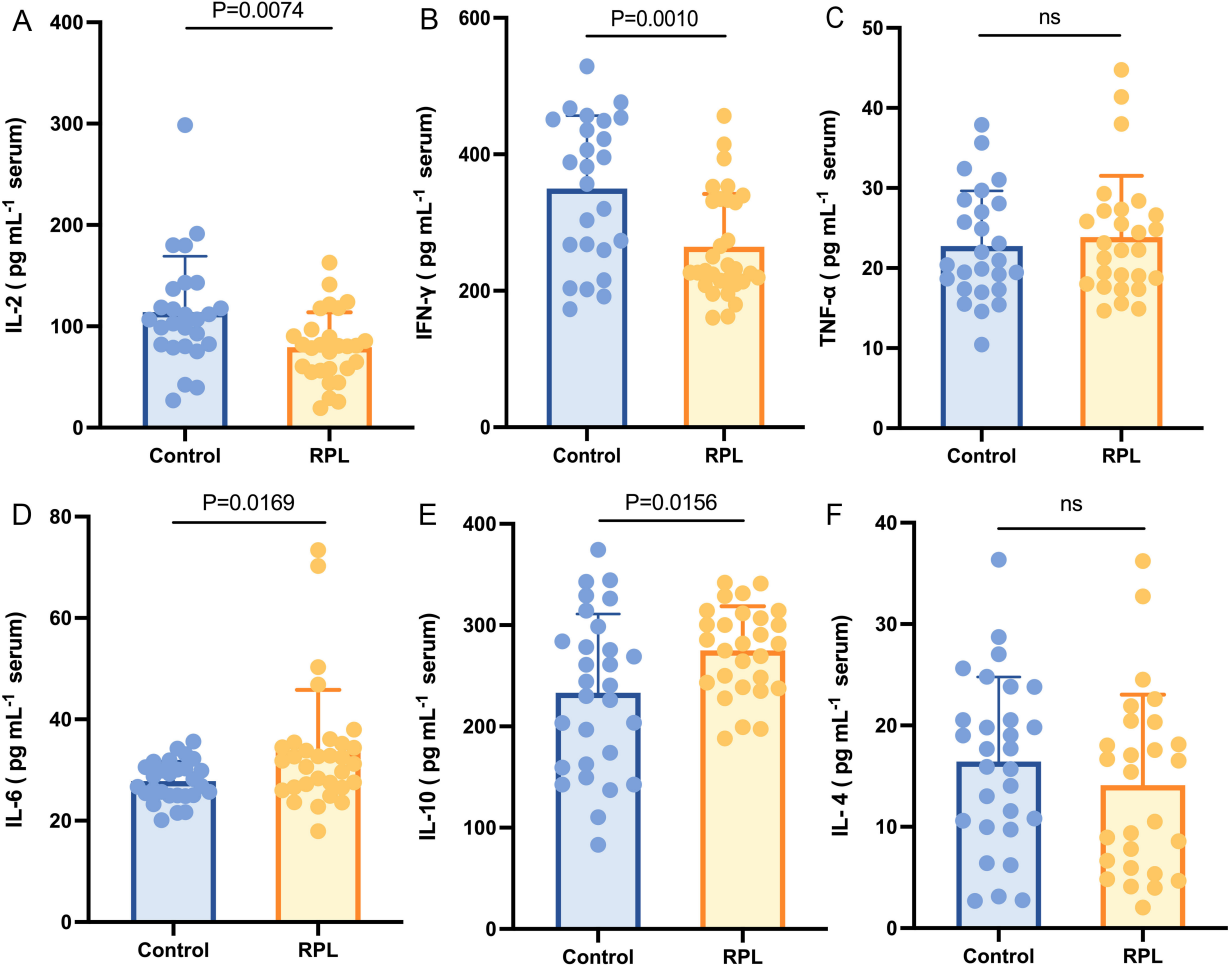
Serum immune cytokines concentration between the RPL (n=34) and control (n=30) groups. Serum IL-2 **(A)** and IFN-γ **(B)** levels were lower in the RPL group (p = 0.007 and 0.001, respectively), while serum IL-6 **(D)** and IL-10 **(E)** levels were higher in the RPL group (p = 0.017 and 0.016, respectively). Serum TNF-α **(C)** and IL-4 **(F)** did not show a significant difference between the groups. RPL, recurrent pregnancy loss; IL-2, interleukin-2; IFN-γ, Interferon-γ; TNF-α, tumor necrosis factor-α; IL-6, interleukin-6; IL-10, interleukin-10; IL-4, interleukin-4. Data presented as mean ± standard deviation.

The average age of the participants was 31.98 ± 3.45 years. The clinical and demographic characteristics of the subjects are listed in **Table 1**. Notably, age and AMH levels did not differ significantly between the groups. However, compared with the control group, serum TSH was lower in the RPL group. The serum concentration of IL-6 (**Figure 1D**) and IL-10 (**Figure 1E**) were significantly higher (p = 0.017 and 0.016, respectively) in the RPL group, while IL-2 (**Figure 1A**) and IFN-γ (**Figure 1B**) levels were significantly lower (p = 0.007 and 0.001, respectively) in the RPL group. No significant differences between the groups were observed in IL-4 (**Figure 1F**) and TNF-α (**Figure 1C**) levels.

**Table 1:** The clinical characteristics and immune cytokines of the RPL and control group.

Variables	RPL	Control	P
Sample numbers	34	30	
Age (y)	31.85 ± 2.79	32.13 ± 4.12	NS
Number of pregnancy losses	2.65 ± 1.10	0	<0.001
AMH (ng/ml)	2.77 ± 1.66	3.26 ± 1.31	NS
TSH (mIU/L)	1.41 ± 0.81	2.24 ± 1.36	0.038
IL-2 (pg/ml)	79.36 ± 34.57	114.01 ± 55.22	0.007
IL-4 (pg/ml)	14.11 ± 8.94	16.44 ± 8.34	NS
IL-6 (pg/ml)	33.71 ± 12.1	27.81 ± 3.95	0.017
IL-10 (pg/ml)	275.13 ± 43.48	233.23 ± 77.87	0.016
TNF-α (pg/ml)	23.86 ± 7.67	22.76 ± 6.88	NS
IFN-γ (pg/ml)	264.7 ± 77.16	349.97 ± 106.59	0.001

RPL, recurrent pregnancy loss; AMH, anti-Müllerian hormone; TSH, thyroid stimulating hormone; IL-2, interleukin-2; IL-4, interleukin-4; IL-10, interleukin-10; IL-6, interleukin-6; IFN-γ, Interferon-γ; TNF-α, tumor necrosis factor-α. Data presented as mean ± standard deviation. P-p value; NS, not significant.

### 3.2 Untargeted metabolomic profiling of serum samples

**Figure 2:**
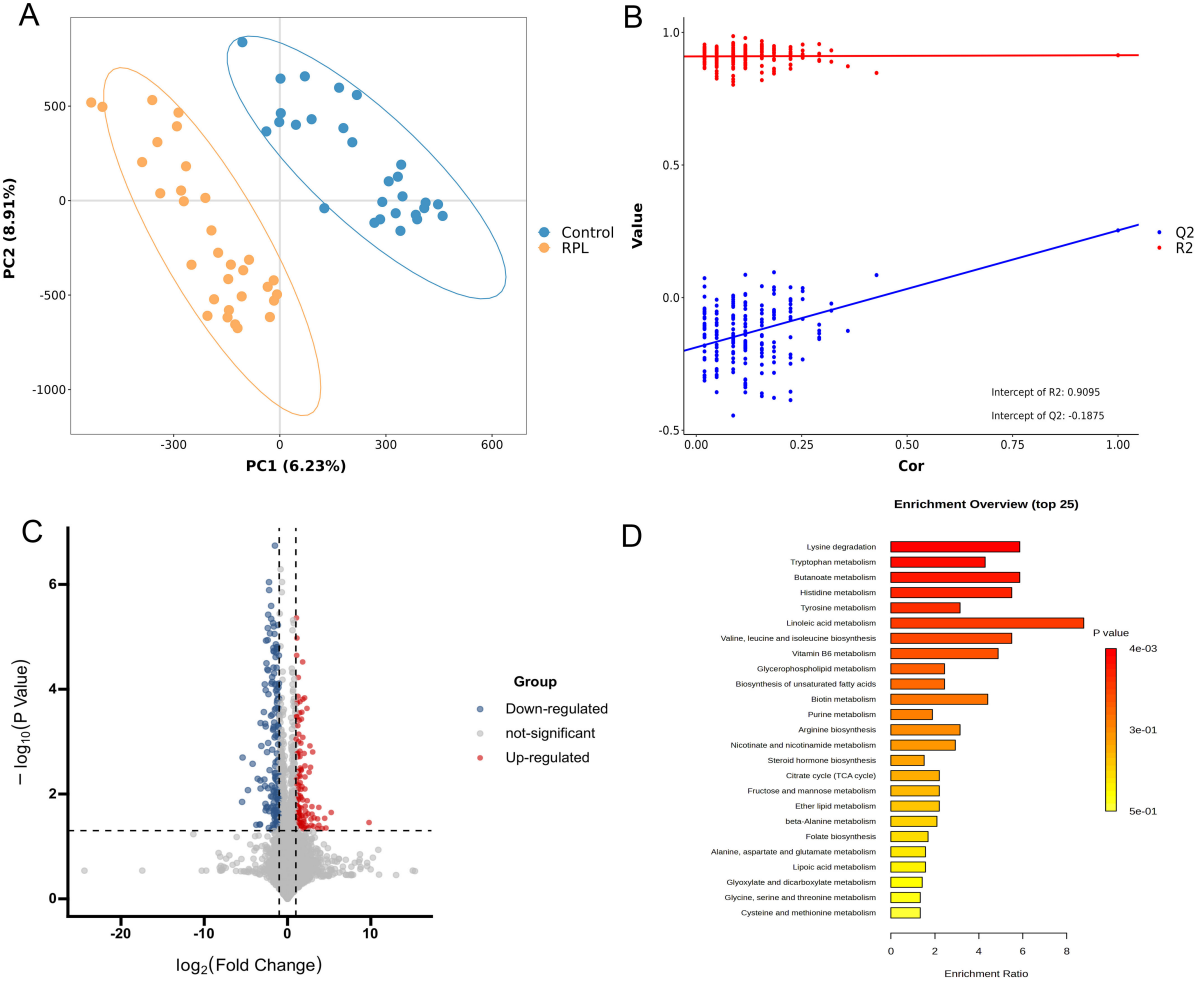
Integrated analysis of untargeted metabolomics data in serum samples between the RPL and control groups. **(A)** PLS-DA score plot in positive and negative polarity mode, **(B)** PLS-DA model permutation test in positive and negative polarity mode, **(C)** volcano plot of 138 significantly increased (red dots) and 158 decreased (blue dots) metabolites in the RPL group, **(D)** the top 25 KEGG pathway enrichment plot of differential metabolites between the RPL and control groups. RPL, recurrent pregnancy loss; PLS-DA, partial least squares discriminant analysis.

In the untargeted metabolomics analysis, to reduce the effects of systematic variation, metabolites with a coefficient of variation > 0.2 in QC samples were removed. Finally, 3184 positive-mode metabolite peaks and 2537 negative-mode metabolite peaks were identified. A clear separation between the serum metabolomic profiles of patients with RPL and the controls was observed in PCA (**Supplementary Figure S1**) and PLS-DA plot under both ionization modes (**Figure 2A**), indicating remarkable metabolic differences between the two groups. The R2 and Q2 values in the PLS‐DA analysis were 0.914 and 0.253, respectively (**Figure 2B**).

To identify significant variances between the groups, metabolites were filtered by both multivariate and univariate statistical criteria (p < 0.05 and FC > 2). The volcano plots illustrated that there were 138 significantly increased and 158 significantly decreased metabolites in the RPL group (**Figure 2C**). Further details on these differential metabolites between the RPL and control groups can be found in **Supplementary Table S1**.

Metabolic pathway analysis was further performed to uncover the disrupted metabolic pathways linked to RPL based on the identified differential metabolites. These metabolites were found to participate in 31 metabolic pathways, prominently in amino acid metabolic pathways such as lysine, branched-chain amino acids, aspartic acid, tryptophan, histidine, tyrosine, arginine, glutamic acid, alanine, cysteine, methionine, serine, glycine and threonine (**Supplementary Table S2**). The top 25 metabolic pathways are depicted in **Figure 2D**. The KEGG pathways showing significant enrichment (p < 0.05) include lysine degradation, tryptophan metabolism, butanoate metabolism, and histidine metabolism. This suggests that amino acid metabolic pathways could potentially have a significant influence on the development of RPL.

### 3.3 Untargeted metabolomic profiles between the subgroup of RPL

**Figure 3:**
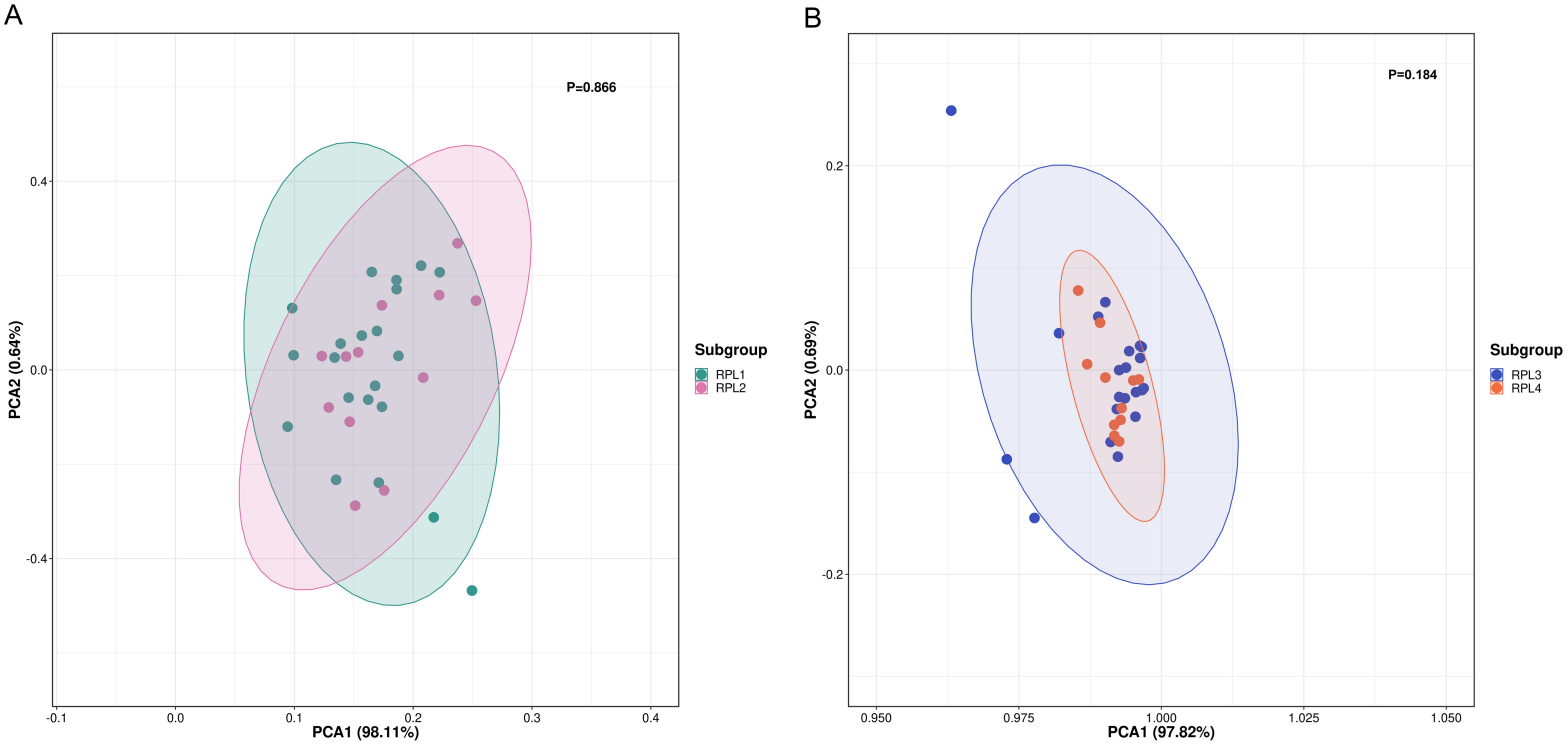
PCA score plot between subgroups of RPL. **(A)** PCA score plot between primary (RPL1, n = 22, green dots) and secondary (RPL2, n = 12, pink dots) RPL (p = 0.866), **(B)** PCA score plot be-tween RPL with two pregnancy losses (RPL3, n = 22, blue dots) and three or more pregnancy losses (RPL4, n = 12, orange dots) (p = 0.184). RPL, recurrent pregnancy loss; PCA, principal component analysis.

RPL is categorized as primary if all prior pregnancies have resulted in pregnancy losses, and as secondary if at least one of the pregnancies progressed beyond 24 weeks or ended with a live birth. Some research suggests that the causes of primary and secondary RPL may be different (18, 19). To investigate the potential influence of primary and secondary RPL, we performed PCA to distinguish metabolite profiles between the two groups. **Figure 3A** demonstrates that there was no difference in the serum metabolome between the primary (n = 22) and secondary (n = 12) RPL groups (p=0.866).

The definition of RPL varies, with some guidelines indicating three or more pregnancy losses, while others suggest two or more losses (1). To explore how the number of pregnancy losses influences serum metabolomic changes, we utilized PCA to differentiate metabolite profiles between groups with two pregnancy losses and those with three or more losses. The findings presented in **Figure 3B** show that the groups with two pregnancy losses (n = 22) were not distinguishable from those with three losses (n = 12) (p = 0.184).

### 3.4 Targeted metabolomics of amino acids in serum samples

**Figure 4:**
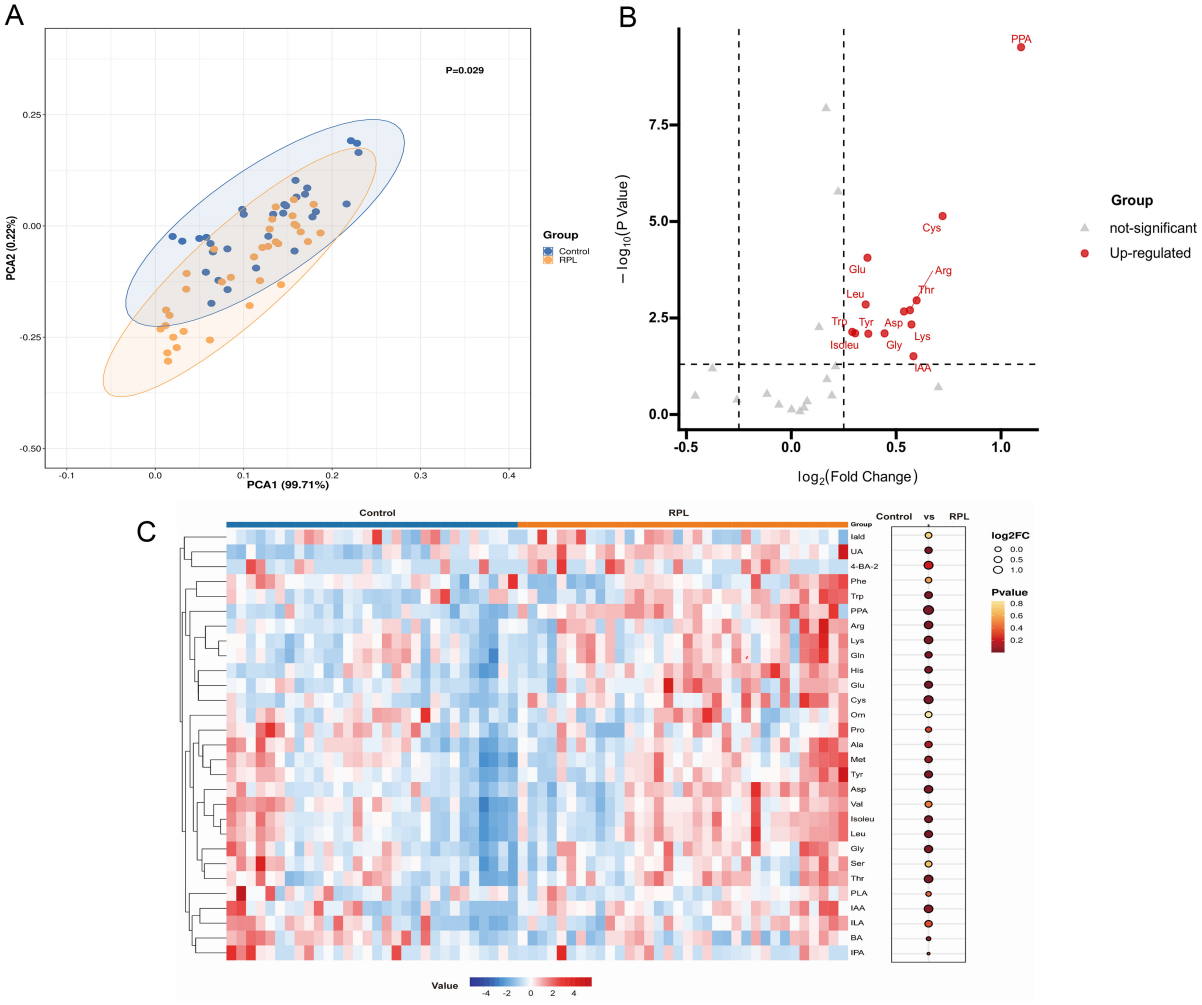
Targeted metabolomic profiling of amino acid metabolites between the RPL and the control group. **(A)** PCA score plot between the RPL (n = 34, yellow dots) and control (n = 30, blue dots) groups (p = 0.029), **(B)** volcano plot of 13 significantly increased (red dots) metabolites in the RPL group, **(C)** heatmap of 29 amino acid metabolites in the RPL and control groups. RPL, re-current pregnancy loss; PCA, principal component analysis; PPA, phenlpyruvic acid; Cys, cystine; Arg, arginine; Thr, threonine; Asp, aspartic acid; Lys, lysine; IAA, indole-3-acetic acid; Gly, glycine; Tyr, tyrosine; Glu, glutamic acid; Leu, leucine; Trp, tryptophan; Isoleu, isoleucine; Iald, indole-3-carboxylic acid; UA, urocaninic acid; 4-BA-2, 4-hydroxybenzoic acid; Phe, phenylalanine; Gln, glntamine; His, histidine; Orn, ornithine; Pro, proline; Ala, alanine; Met, methionine; Val, valine; Ser, serine; PLA, phenyllactic acid; ILA, Indole-3-lactic acid; BA, benzoic acid; IPA, indole-3-propionic acid.

In untargeted metabolomics analysis, we observed a notable differentiation in amino acid metabolism pathways between the RPL and control groups. Subsequently, we conducted a targeted metabolomics analysis focusing on amino acid metabolism-related metabolites, covering 43 amino acid metabolites, of which 29 were detected. PCA analysis was first performed to reveal distinct discrepancies between the RPL and control groups (P=0.029) (**Figure 4A**). The volcano plots highlighted 13 amino acid metabolites that increased in the RPL group compared to the control group (**Figure 4B**). Notably, IAA, tyrosine, glycine, isoleucine, tryptophan, lysine, aspartic acid, arginine, leucine, threonine, glutamic acid, cystine, and PPA were up-regulated in serum samples of the RPL group (**Figure 4C**). These results confirm changes in amino acid metabolism during the development of RPL.

### 3.5 Discovery of diagnostic biomarkers by random forest model and ROC analysis

**Figure 5:**
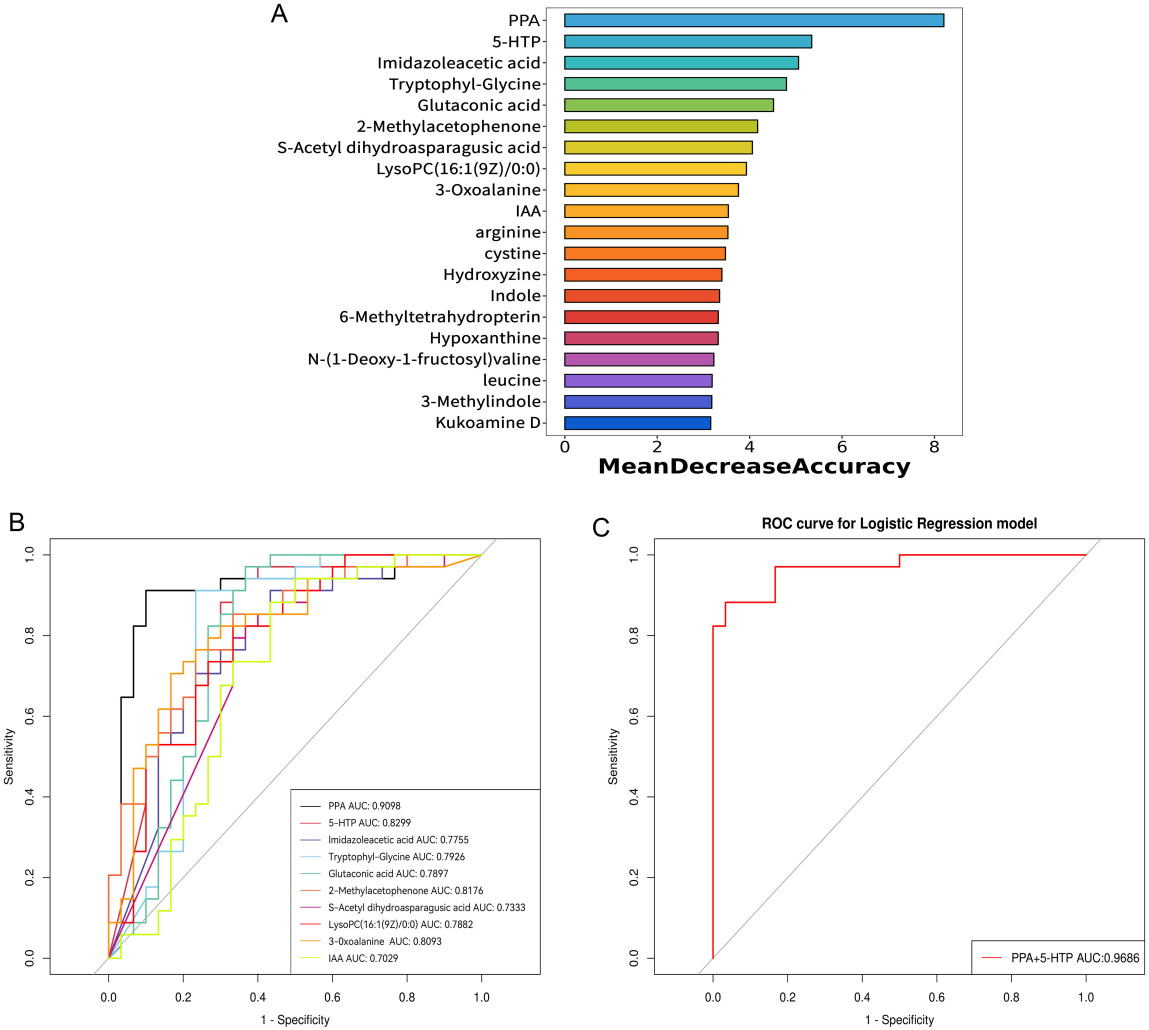
Identification of potential serum metabolite biomarkers of RPL. **(A)** the top 20 metabolites with high contribution in the random forest model, **(B)** ROC curve and AUC value of the top 10 metabolites identified in the random forest model, **(C)** ROC curve and AUC value for the combined top 2 metabolites identified in the random forest model. RPL, recurrent pregnancy loss; PPA, phenlpyruvic acid; IAA, indole-3-acetic acid; 5-HTP, 5-hydroxy-L-tryptophan; ROC, receiver operating characteristic; AUC, area under the ROC curve.

To improve the ability to distinguish, a random forest model was constructed using clinical indicators, immune cytokines, and all differential serum metabolites between groups as input data. The model included the top 20 metabolites with the highest contributions, as illustrated in **Figure 5A**. Subsequently, ROC curves were created and the area under the ROC curve (AUC) was determined to assess the capability of these top 10 metabolites as biomarkers for RPL. The AUC values for PPA, 5-hydroxy-L-tryptophan (5-HTP), imidazoleacetic acid, tryptophyl-glycine, glutaconic acid, 2-methylacetophenone, S-acetyl dihydroasparagusic acid, lysoPC(16:1(9Z)/0:0), 3-oxoalanine, and IAA were 0.910, 0.830, 0.776, 0.793, 0.790, 0.818, 0.733, 0.788, 0.809, and 0.703, respectively (**Figure 5B**). Subsequently, a diagnostic model based on these top 2 metabolites, PPA and 5-HTP, was developed. Evaluation of the model’s diagnostic performance using a ROC curve (**Figure 5C**) revealed an impressive AUC value of 0.969. Overall, PPA and 5-HTP showed potential as predictive markers for RPL.

### 3.6 Correlation analysis between differential metabolites and immune cytokines

**Figure 6:**
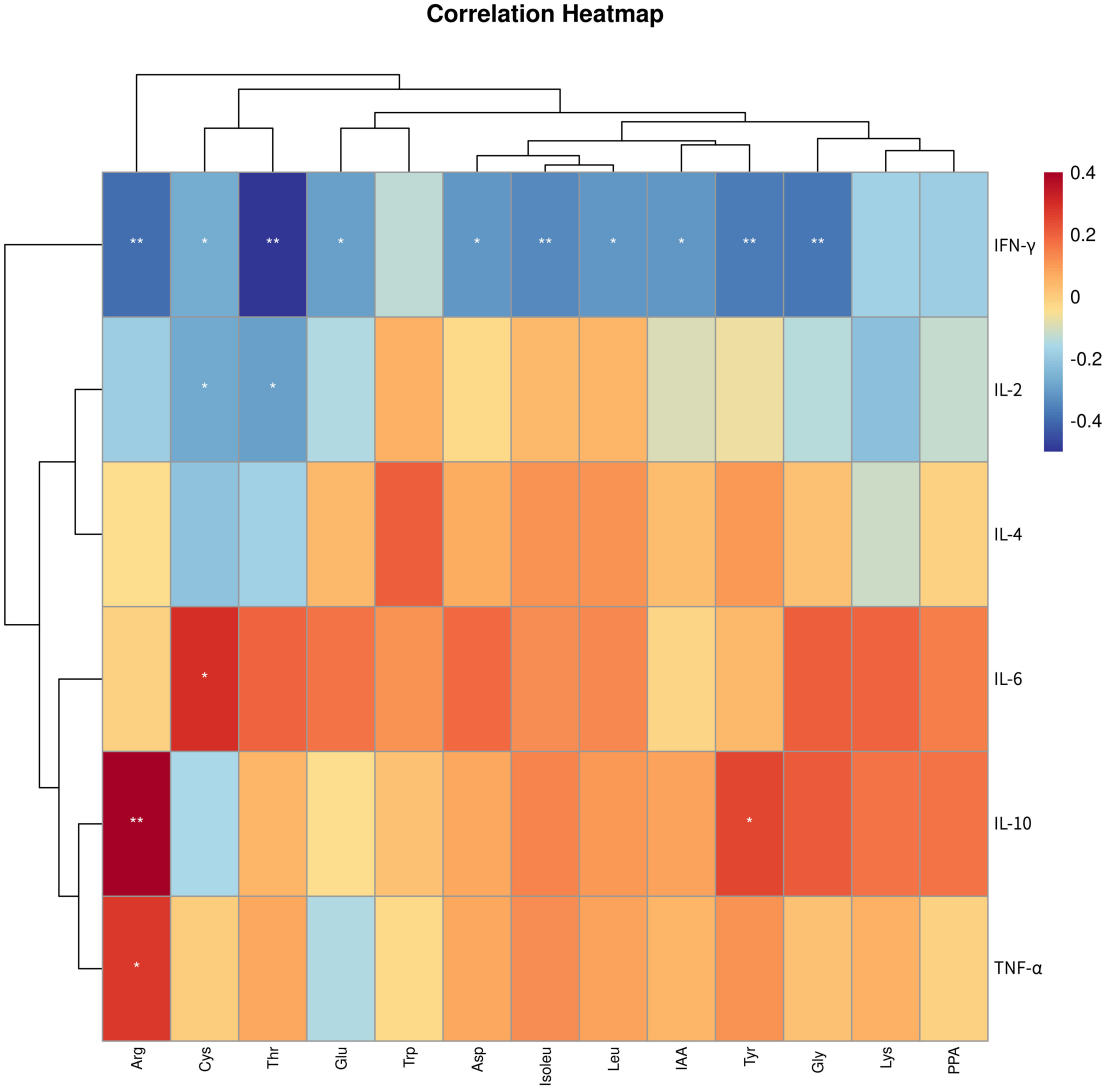
Correlation analysis between differential amino acid metabolites and immune cytokines. * p < 0.05, ** p < 0.01. IFN-γ, Interferon-γ; IL-2, interleukin-2; TNF-α, tumor necrosis factor-α; IL-4, interleukin-4; IL-10, interleukin-10; IL-6, interleukin-6; PPA, phenlpyruvic acid; Cys, cystine; Arg, arginine; Thr, threonine; Asp, aspartic acid; Lys, lysine; IAA, indole-3-acetic acid; Gly, glycine; Tyr, tyrosine; Glu, glutamic acid; Leu, leucine; Trp, tryptophan; Isoleu – isoleucine.

To clarify the connection between metabolites and the disease, we conducted Spearman correlation analysis to explore the connections between the 13 differential metabolites identified through targeted metabolomics analysis and immune cytokines. As depicted in **Figure 6**, our findings indicated that arginine, cystine, threonine, glutamic acid, aspartic acid, isoleucine, leucine, IAA, tyrosine, and glycine showed negative associations with IFN-γ, while cystine and threonine exhibited negative associations with IL-2. Additionally, cystine displayed a positive correlation with IL-6, while arginine and tyrosine were positively correlated with IL-10, and arginine showed a positive correlation with TNF-α.

## 4 Discussion

Identifying biomarkers and understanding the pathophysiological processes of RPL are essential for its prevention and treatment. It is commonly acknowledged that immune dysregulation plays a significant role in RPL (2, 20). In this study, we examined Th1/Th2-related immune cytokines and found significant differences in the levels of IL-6, IL-10, IL-2, and IFN-γ between the RPL and control groups. Additionally, untargeted and targeted metabolomics analyses indicated notable variances in amino acid metabolites between the two groups. Nevertheless, we did not detect any differential metabolites between the primary and secondary RPL groups, nor within subgroups of RPL categorized by the number of pregnancy losses. A diagnostic model highlighted PPA and 5-HTP as potential biomarkers for RPL. Correlation analysis revealed associations between amino acid metabolites and immune function.

Multiple studies have indicated that dysregulation of Th1/Th2 immunity may contribute to RPL (5, 21, 22). Th1 cells participate in cellular immunity by generating proinflammatory cytokines such as IL-2, TNF-α, and IFN-γ, while Th2 cells release anti-inflammatory cytokines like IL-4, IL-6, and IL-10, and play a role in humoral immunity (23). Our findings revealed elevated levels of IL-6 and IL-10, and decreased levels of IL-2 and IFN-γ in the RPL group, with no significant variations in TNF-α and IL-4 levels between the groups. These results indicate a dysregulation of Th1/Th2 immunity and a prevalence of Th2 immunity in nonpregnancy individuals with RPL. Several studies have compared blood immune cytokines in RPL patients to those in healthy controls, with some findings aligning with our results and others showing discrepancies (24–27). These variations may stem from differences in the stages of reproduction among the patients included in the studies. Cytokines play a crucial role in all stages of reproduction and exhibit dynamic changes throughout the process (28, 29). The importance of our research lies in offering references and evidence to assess whether individuals are at a heightened risk of RPL based on the detection of cytokines.

Prior studies have suggested a noticeable differentiation in the causes of RPL between primary and secondary RPL (19, 30). However, recent research has indicated that there is no substantial variance in the underlying causes of primary and secondary RPL (31). Through untargeted metabolomic profiling, it was found that there are no variations in serum metabolites between primary and secondary RPL patients. Similarly, there is ongoing debate regarding whether there is a disparity in the etiology between three or more pregnancy losses and those with two losses (32, 33). Our study reveals that there is no significant distinction in serum metabolites between individuals with three or more pregnancy losses and those with two losses through untargeted metabolomic profiling. These results provide metabolomics-based evidence supporting the idea that there are no significant distinctions in the etiologies of primary and secondary RPL, as well as RPL between individuals with three or more pregnancy losses and those with two losses.

Metabolomics captures the alterations in the body resulting from genetic and environmental influences (34). In the presence of disease, cells, tissues, and organisms exhibit specific metabolic responses, altering the profile, composition, and levels of endogenous metabolites. Through metabolomics, these shifts can be identified, offering a distinctive approach to uncovering disease mechanisms and identifying biomarkers linked to various conditions (34–36). Our metabolomics analysis revealed significant metabolic distinctions between the RPL and control cohorts. Certain metabolites could serve crucial roles in diagnosing or predicting RPL, as well as contributing to its pathogenesis. PPA, an intermediate in the phenylalanine metabolic pathway, is of particular interest. Elevated levels of PPA can impact nerve cell proliferation, leading to abnormal fetal nervous system development (37, 38). This compound also hinders the metabolism of tetrahydrobiopterin, whose deficiency can disrupt uterine placental remodeling, resulting in fetal growth restriction and pregnancy-related hypertension (39, 40). Additionally, studies suggest that PPA may trigger excessive activation of nucleotide-binding oligomerization domain-like receptor protein 3 inflammasomes, exacer bating inflammatory processes (41). Based on the available evidence, it is suggested that PPA may play a role in vascular remodeling and inflammation, both key pathogenic mechanisms in RPL. Currently, no research has directly reported the involvement of PPA in the pathogenesis of RPL. Further investigation into the role of PPA in placental angiogenesis and immune regulation within the endometrium in RPL could be a promising area of study. 5-HTP is derived from tryptophan and serves as a precursor to serotonin. Studies suggest that 5-HTP is linked to emotions, appetite, and physical activity (42, 43). Moreover, supplementing with 5-HTP can increase serotonin levels in the body, influencing the regulation of immune cells and immune factor release (44). Our study indicates that 5-HTP may be a valuable biomarker for RPL, potentially due to the immunomodulatory effects of its metabolite serotonin. Further investigation is required to elucidate the specific mechanisms involved. Among the top 10 metabolites identified by the random forest algorithm, including imidazoleacetic acid, tryptophyl-glycine, glutaconic acid, 2-methylacetophenone, S-acetyl dihydroasparagusic acid, lysoPC(16:1(9Z)/0:0), 3-oxoalanine, and IAA, there is limited research on their association with RPL. Further investigation is necessary to elucidate their potential roles in RPL.

Our findings suggest notable abnormalities in amino acid metabolism in RPL, aligning with findings from various existing studies. These abnormalities were identified in both the blood and decidua of RPL patients (11, 45, 46). Previous literature has highlighted that disrupted amino acid metabolism can hinder maternal decidualization and increase the risk of RPL (45, 47). Additionally, the expression of amino acid transporters at the maternal-fetal interface differs between RPL patients and healthy controls, including SLC3A2 and SLC7A11 (48, 49). These differences in amino acid transporter expression contribute to metabolic changes in amino acids that are involved in RPL pathogenesis. Besides, the primary pathogenesis of RPL involves immune dysfunction, with recent research indicating that amino acid metabolism contributes to the regulation of immune function. Amino acids serve as essential nutrients for immune cells throughout tissue maintenance, organ development, and immune responses. Changes in amino acid metabolism are crucial in modulating the differentiation and function of immune cells (50).

In our correlation analysis between differential amino acid metabolites and immune cytokines, we observed that cystine and tyrosine showed a positive correlation with Th2 cytokines and a negative correlation with Th1 cytokines. In our study, we observed elevated levels of cystine in the RPL group, with a positive correlation with the Th2 cytokine IL-6 and negative correlations with the Th1 cytokines IFN-γ and IL-2. Cystine, a non-essential amino acid derived from two cysteine molecules, is involved in protein synthesis, glutathione production, immune function modulation, and maintaining intracellular redox equilibrium (51, 52). The proliferation and activation of T cells rely on a delicate balance of intracellular oxidative and reducing agents. Key among these are cysteine and glutathione, which are produced by cystine within cells. However, immature T cells lack the expression of cysteine transporters. Studies have shown that antigen-presenting cells and macrophages can convert transported cysteine into cysteine within cells, regulating its delivery to T lymphocytes in a controlled manner. This process plays a role in modulating T lymphocyte differentiation (53, 54). Research has shown that supplementing with cysteine can boost the Th2 response (55). This aligns with our findings, showing a positive correlation between elevated levels of cystine and the Th2 cytokine IL-6. The upsurge in cystine levels may play a role in the onset of RPL by prompting the differentiation of Th cells into Th2 cells. Our findings indicated a significant rise in tyrosine levels in RPL compared to the control group, aligning with previous studies on serum metabolomics during the implantation window period of RPL (11). The increase in tyrosine levels was negatively related to the Th1 cytokine IFN-γ and positively linked to the Th2 cytokine IL-10. Tyrosine can be transformed into vital compounds such as dopamine, norepinephrine, epinephrine, and thyroid hormones in humans, playing a vital role in the nervous system (56). While there are no existing literature reports on the interaction between tyrosine and Th cells, further investigation is needed to understand its role in RPL. Arginine exhibits differential expression in RPL, in line with earlier findings (8, 11). Arginine serves as a critical regulator of immune cell activities, with T cells being particularly responsive to changes in their levels (57, 58). Our study revealed associations between arginine and the immune cytokines TNF-α, IL-10, and IFN-γ. Additional research is needed to clarify the potential involvement of arginine in the pathogenesis of RPL through immune modulation. Moreover, our analysis revealed correlations between threonine, glutamic acid, aspartic acid, isoleucine, leucine, IAA, glycine, and immune cytokines. The impact of increased levels of these amino acid metabolites on the development of RPL requires further investigation.

We acknowledge the limitations of this study. Firstly, it was a cross-sectional research, thus, the cause-and-effect relationship between metabolites and RPL could not be determined. A prospective study with clinical intervention is recommended to address this uncertainty. Secondly, the number of participants in our study was relatively small, and it is advisable to validate our findings in larger cohorts. Additionally, the diagnostic model and potential mechanisms identified in our study have not been validated or further investigated. Lastly, our research focused on patients already diagnosed with RPL, which may not be representative of individuals with a predisposition to RPL but no history of pregnancy loss. Nevertheless, we believe that this metabolomic analysis of RPL will offer valuable insights for the establishment of diagnostic criteria for individuals at risk of RPL.

## 5 Conclusion

In summary, our study underscores the significance of amino acid metabolism in developing RPL. It indicates that PPA and 5-HTP could serve as promising predictive indicators for RPL, while cysteine and tyrosine might have pivotal roles in the immune response linked to RPL. A deeper exploration of the underlying molecular mechanisms could offer novel insights for advancing diagnostic and therapeutic approaches for RPL.

## Data Availability

The raw data supporting the conclusions of this article will be made available by the authors, without undue reservation.
