# Supplementary material for: Metabolomic analysis reveals potential role of immunometabolism dysregulation in recurrent pregnancy loss
Source: Front Endocrinol (Lausanne). 2024 Oct 9;15:1476774. doi: 10.3389/fendo.2024.1476774 (PMC11496058; doi:10.3389/fendo.2024.1476774)
Supplement: Supplementary file 1 [file DataSheet1.docx]

Supplementary Material

# Supplementary Figure

**
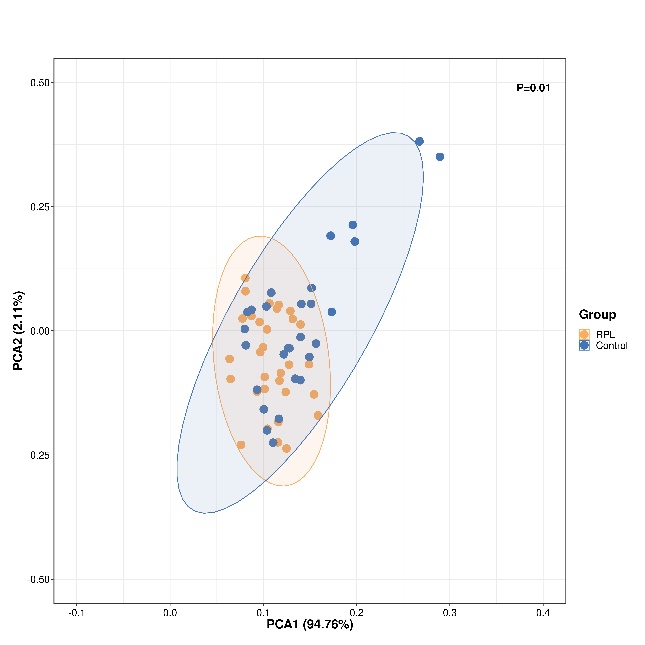
**

**Supplementary Figure 1.** PCA score plot between RPL (n = 34, yellow dots) and control (n = 30, blue dots) group (p = 0.01) in untargeted metabolomics data

# Supplementary Tables

**Supplementary Table 1.** Differential metabolites in serum between the RPL (n = 34) and control (n = 30) groups

| Compound | Compound ID | Description | log2(FC) | VIP | P.Value |
| --- | --- | --- | --- | --- | --- |
| 0.69_145.0978m/z | HMDB0000182 | L-Lysine | 1.304377 | 1.38645 | 0.012539 |
| 0.69_154.0618m/z | HMDB0000177 | L-Histidine | 1.112636 | 1.83939 | 0.000176 |
| 0.69_168.0771m/z | HMDB0002024 | Imidazoleacetic acid | -1.51989 | 1.89544 | 5E-05 |
| 0.70_189.1601m/z | HMDB0001325 | N6,N6,N6-Trimethyl-L-lysine | 1.215165 | 1.12896 | 0.049239 |
| 0.70_212.0410m/z | HMDB0035717 | S-Acetyl dihydroasparagusic acid | -1.75838 | 1.74353 | 0.000322 |
| 0.70_327.0514m/z | HMDB0060641 | Lamivudine-monophosphate | 1.088997 | 1.54623 | 0.009313 |
| 0.73_1183.7787m/z | HMDB0075588 | CL(i-14:0/i-12:0/i-12:0/i-14:0) | -1.16178 | 1.36707 | 0.012713 |
| 0.73_902.3125m/z | HMDB0015066 | Methacycline | 3.789781 | 1.21026 | 0.018011 |
| 0.75_1180.7147m/z | HMDB0116132 | CDP-DG(a-21:0/i-24:0) | -3.49712 | 1.79735 | 0.018347 |
| 0.76_156.0423m/z | HMDB0060173 | N-Methylethanolaminium phosphate | 1.354682 | 1.92731 | 0.000721 |
| 0.76_166.0845m/z | HMDB0004827 | Proline betaine | 1.750855 | 1.18883 | 0.040885 |
| 0.76_182.0583m/z | HMDB0001190 | Indoleacetaldehyde | 3.239113 | 1.23982 | 0.028491 |
| 0.77_214.1306m/z | HMDB0014685 | Pyridostigmine | -1.12966 | 1.45467 | 0.00821 |
| 0.77_816.3949m/z | HMDB0014516 | Thiethylperazine | 2.344062 | 1.21255 | 0.039565 |
| 0.78_130.0863m/z | HMDB0000039 | Butyric acid | 1.527803 | 1.31019 | 0.042677 |
| 0.78_187.0583m/z | HMDB0000174 | L-Fucose | 1.288394 | 1.45471 | 0.042677 |
| 0.79_316.1357m/z | HMDB0039780 | N-(1-Deoxy-1-fructosyl)isoleucine | 1.549827 | 1.57062 | 0.006061 |
| 0.80_663.2869m/z | HMDB0038449 | Vomifoliol 9-[glucosyl-(1->4)-xylosyl-(1->6)-glucoside] | 2.060926 | 2.11598 | 0.000146 |
| 0.80_685.2696m/z | HMDB0030107 | 3-Hydroxy-3-(3,4-dihydroxy-4-methylpentanoyl)-5-(3-methylbutyl)-1,2,4-cyclopentanetrione | 1.238826 | 1.42754 | 0.018678 |
| 0.81_110.0350m/z | HMDB0060659 | 1-Methyl-4-nitroimidazole | 3.281958 | 1.22266 | 0.041552 |
| 0.81_130.0868m/z | HMDB0000687 | L-Leucine | 1.813787 | 1.42674 | 0.011718 |
| 0.81_189.1022m/z | HMDB0005785 | Indole-3-carbinol | -1.12912 | 1.99283 | 0.000819 |
| 0.81_350.0983m/z | HMDB0012226 | Entacapone | 1.949476 | 1.48942 | 0.012965 |
| 0.81_357.0306m/z | HMDB0061052 | 4-hydroxytriazolam | 1.01779 | 1.53655 | 0.001826 |
| 0.82_335.0487m/z | HMDB0000289 | Uric acid | 1.822732 | 1.64212 | 0.008179 |
| 0.83_242.0306m/z | HMDB0006955 | 3-Hydroxy-2-methylpyridine-4,5-dicarboxylate | -1.13477 | 2.09075 | 5.99E-06 |
| 0.84_167.0468m/z | HMDB0061890 | Pyroglutamylglycine | -1.13355 | 2.23232 | 2.28E-05 |
| 0.94_376.1498m/z | HMDB0000714 | Hippuric acid | 1.257589 | 1.45635 | 0.016709 |
| 0.95_310.1287m/z | HMDB0037846 | N-(1-Deoxy-1-fructosyl)phenylalanine | 1.233145 | 1.53924 | 0.01243 |
| 0.96_712.4086m/z | HMDB0038827 | Murrayazolinol | 1.639755 | 1.30081 | 0.022443 |
| 0.97_122.0482n | HMDB0001406 | Niacinamide | 2.655572 | 1.17487 | 0.043828 |
| 0.97_167.0455m/z | HMDB0061890 | Pyroglutamylglycine | -1.04701 | 1.92394 | 0.000376 |
| 0.97_201.0732m/z | HMDB0033789 | 2-O-Methyl-L-fucose | 1.826242 | 2.31247 | 3.02E-05 |
| 0.97_338.1439m/z | HMDB0041339 | 5-Chloro-2-(3,5-di-tert-butyl-2-hydroxyphenyl)-2H-benzotriazole | -2.43545 | 1.17203 | 0.018257 |
| 0.99_381.0961m/z | HMDB0040556 | 5-Hydroxyflavone | 4.984555 | 1.13903 | 0.044615 |
| 0.99_499.0738m/z | HMDB0000797 | SAICAR | 2.389858 | 1.92536 | 0.000232 |
| 1.02_172.0717m/z | HMDB0015052 | Metronidazole | 10.11778 | 1.28728 | 0.034985 |
| 1.03_165.0545m/z | HMDB0000118 | Homovanillic acid | -1.57336 | 1.88561 | 0.000565 |
| 1.03_194.0811m/z | HMDB0001434 | 3-Methoxytyrosine | -1.44805 | 2.00191 | 0.000243 |
| 1.03_238.0447m/z | HMDB0000114 | Glycerylphosphorylethanolamine | -1.53517 | 1.95642 | 0.000431 |
| 1.05_243.1190m/z | HMDB0014925 | Aciclovir | 1.787268 | 1.92611 | 0.000372 |
| 1.06_148.0760m/z | HMDB0000022 | 3-Methoxytyramine | -1.74434 | 2.03922 | 5.85E-05 |
| 1.06_192.0658m/z | HMDB0011723 | 2-Methylhippuric acid | -2.332 | 2.16015 | 1.15E-05 |
| 1.06_219.0767m/z | HMDB0000472 | 5-Hydroxy-L-tryptophan | -2.33776 | 2.3225 | 3.78E-06 |
| 1.06_277.0356m/z | HMDB0029318 | Caffeoylmalic acid | -1.10998 | 1.94148 | 0.000304 |
| 1.06_287.0642m/z | HMDB0059600 | 5-phosphonooxy-L-lysine | -2.26852 | 2.04595 | 4.38E-05 |
| 1.06_309.0464m/z | HMDB0033622 | Cabbage identification factor 2 | -1.68519 | 2.22369 | 5.45E-06 |
| 1.06_434.9910m/z | HMDB0015284 | Sertaconazole | -1.25058 | 1.66596 | 0.001689 |
| 1.06_715.3675m/z | HMDB0060164 | Neocasomorphin | 1.102396 | 1.82161 | 0.000333 |
| 1.07_456.2379m/z | HMDB0037039 | 4-O-Methylmelleolide | 2.007322 | 1.81313 | 0.00107 |
| 1.08_757.3984m/z | HMDB0034327 | Glucoconvallasaponin B | 1.327742 | 1.84666 | 0.000497 |
| 1.11_180.0804m/z | HMDB0035400 | 1-Tridecene-3,5,7,9,11-pentayne | 3.039995 | 1.60396 | 0.003087 |
| 1.11_331.0904m/z | HMDB0001886 | 3-Methylxanthine | 1.273132 | 1.36537 | 0.01309 |
| 1.11_801.4241m/z | HMDB0036244 | Schidigerasaponin F2 | 1.53613 | 1.96848 | 0.000173 |
| 1.12_321.0615m/z | HMDB0126548 | 3,4,5-trihydroxy-6-(3-phenyloxirane-2-carbonyloxy)oxane-2-carboxylic acid | 1.096034 | 1.13668 | 0.036008 |
| 1.12_981.5199m/z | HMDB0038569 | PC-M5' | 1.747153 | 1.94163 | 0.000394 |
| 1.13_1025.5458m/z | HMDB0059791 | Adrenorphin | 1.439769 | 1.43787 | 0.021084 |
| 1.13_320.1325m/z | HMDB0000403 | 2-Hydroxyadenine | 2.012534 | 1.73291 | 0.001684 |
| 1.14_215.1250m/z | HMDB0014989 | Dacarbazine | -1.38718 | 1.32592 | 0.009116 |
| 1.16_597.3129m/z | HMDB0035233 | Olomoucine | 1.164319 | 2.48632 | 1.06E-05 |
| 1.17_195.9129m/z | HMDB0039853 | 3,5,6-Trichloro-2-pyridinol | -1.05684 | 1.16608 | 0.04639 |
| 1.17_351.0624m/z | HMDB0014544 | Griseofulvin | -1.79439 | 1.50567 | 0.007527 |
| 1.18_291.0566m/z | HMDB0031783 | Etrimfos | -1.33591 | 1.90259 | 0.000106 |
| 1.18_306.1421m/z | HMDB0028718 | Arginyl-Serine | -2.29681 | 1.42804 | 0.007751 |
| 1.18_333.1538m/z | HMDB0028704 | Arginyl-Asparagine | -2.00515 | 1.94264 | 0.000305 |
| 1.18_359.0440m/z | HMDB0130069 | {[1-hydroxy-1-(1-oxo-1H-isochromen-3-yl)butan-2-yl]oxy}sulfonic acid | -1.33922 | 2.10658 | 1.72E-05 |
| 1.18_365.1251m/z | HMDB0132999 | 3,4,5-trihydroxy-6-{[5-(4-methoxyphenyl)-3-oxopentan-2-yl]oxy}oxane-2-carboxylic acid | -2.56059 | 1.27533 | 0.017535 |
| 1.19_160.0432m/z | HMDB0031340 | Cyclamic acid | -2.99667 | 1.58216 | 0.001213 |
| 1.19_255.0414m/z | HMDB0013870 | cis,trans-5'-Hydroxythalidomide | -1.4883 | 2.03037 | 8.22E-05 |
| 1.19_641.3346m/z | HMDB0041824 | alpha-Zearalenol | 1.272759 | 2.18153 | 0.000137 |
| 1.21_354.1741m/z | HMDB0005012 | Olanzapine | 1.084173 | 2.38892 | 2.26E-05 |
| 1.23_987.5602m/z | HMDB0116805 | CL(8:0/8:0/8:0/14:0) | 2.322322 | 1.64607 | 0.002903 |
| 1.24_496.2377m/z | HMDB0000038 | Dihydrobiopterin | 1.304918 | 1.55415 | 0.003698 |
| 1.25_165.0190m/z | HMDB0001866 | 3,4-Dihydroxymandelic acid | 2.827804 | 1.26395 | 0.006183 |
| 1.26_361.0770m/z | HMDB0128036 | 3,4,5-trihydroxy-6-(2-hydroxy-3-methoxyphenoxy)oxane-2-carboxylic acid | 2.395428 | 1.35019 | 0.013413 |
| 1.27_321.1310m/z | HMDB0011599 | 1-Methyladenine | 3.639071 | 1.18325 | 0.029837 |
| 1.30_203.0823m/z | HMDB0000929 | L-Tryptophan | -1.10755 | 1.16586 | 0.017653 |
| 1.32_475.2427m/z | HMDB0010341 | Dextrorphan O-glucuronide | 1.580146 | 1.4905 | 0.010892 |
| 1.35_628.3214m/z | HMDB0005773 | Endomorphin-1 | 4.847636 | 1.36361 | 0.022519 |
| 1.37_504.2616m/z | HMDB0005777 | Morphiceptin | 1.758397 | 1.99277 | 0.000275 |
| 1.39_513.2784m/z | HMDB0059773 | S-3-oxodecanoyl cysteamine | 1.064663 | 2.39509 | 4.35E-06 |
| 1.41_115.1000n | HMDB0031651 | N-(3-Methylbutyl)acetamide | -1.19424 | 1.77138 | 0.001128 |
| 1.42_383.1529m/z | HMDB0062544 | 16a-hydroxy DHEA 3-sulfate | 1.462391 | 1.20204 | 0.027976 |
| 1.49_129.0583n | HMDB0011664 | 3-Methylene-indolenine | -1.07918 | 1.72871 | 0.002393 |
| 1.49_142.0868m/z | HMDB0000062 | L-Carnitine | -1.12636 | 2.09341 | 0.000345 |
| 1.49_169.0977m/z | HMDB0000446 | N-Alpha-acetyllysine | -1.05024 | 2.07035 | 0.000165 |
| 1.49_229.0536m/z | HMDB0032401 | (+/-)-3-[(2-methyl-3-furyl)thio]-2-butanone | -1.26744 | 1.85509 | 0.000708 |
| 1.49_261.0609m/z | HMDB0000620 | Glutaconic acid | -1.46402 | 2.29431 | 2.44E-05 |
| 1.49_295.0441m/z | HMDB0029421 | N-Acetyldjenkolic acid | -1.18592 | 1.29855 | 0.012289 |
| 1.49_299.0556m/z | HMDB0001587 | Phenylglyoxylic acid | -1.53791 | 1.52202 | 0.004941 |
| 1.49_321.1423m/z | HMDB0060654 | Citalopram N-oxide | -1.28812 | 1.3328 | 0.005202 |
| 1.49_361.1853m/z | HMDB0002249 | 6-Methyltetrahydropterin | -2.81664 | 1.89497 | 0.000455 |
| 1.49_373.0601m/z | HMDB0128985 | {[4-(7-methoxy-2-oxo-2H-chromen-6-yl)butan-2-yl]oxy}sulfonic acid | -1.44851 | 2.19668 | 1.39E-05 |
| 1.49_441.0475m/z | HMDB0128488 | 6-({13,14-dihydroxy-9-oxo-8,17-dioxatetracyclo[8.7.0.0虏,鈦?0鹿鹿,鹿鈦禲heptadeca-1(10),2(7),3,5,11(16),12,14-heptaen-5-yl}oxy)-3,4,5-trihydroxyoxane-2-carboxylic acid | -1.74549 | 2.17983 | 1.63E-05 |
| 1.49_458.0383m/z | HMDB0059599 | CDP-glycerol | -1.99359 | 1.44779 | 0.008809 |
| 1.50_289.0464m/z | HMDB0038641 | 1-[4,9-Dihydro-2-(methylthio)-1,3-thiazino[6,5-b]indol-4-yl]-2-propanone | -1.76714 | 2.09636 | 3.86E-05 |
| 1.50_497.1598m/z | HMDB0030495 | Artonol D | -4.14489 | 1.70408 | 0.002655 |
| 1.51_251.0528m/z | HMDB0028781 | Cysteinyl-Methionine | 1.335461 | 1.2835 | 0.039954 |
| 1.51_264.0123n | HMDB0038142 | 5,8-Dihydro-6-(4-methyl-3-pentenyl)-1,2,3,4-tetrathiocin | 1.349402 | 1.39962 | 0.017026 |
| 1.52_205.0476m/z | HMDB0011602 | 3-Oxoalanine | 1.416579 | 1.63847 | 0.003689 |
| 1.52_409.0090m/z | HMDB0014777 | Butoconazole | 1.406738 | 1.81293 | 0.001656 |
| 1.61_130.0654m/z | HMDB0005785 | Indole-3-carbinol | -1.27548 | 2.06346 | 0.000183 |
| 1.61_132.0811m/z | HMDB0000466 | 3-Methylindole | -1.23442 | 2.08167 | 0.000164 |
| 1.61_159.0919m/z | HMDB0000259 | Serotonin | -1.23519 | 2.11833 | 0.000124 |
| 1.61_178.0863m/z | HMDB0032603 | 2-Hydroxy-4-methylbenzaldehyde | -1.13993 | 2.08942 | 0.000153 |
| 1.62_105.0703m/z | HMDB0032619 | 1-Phenylethanol | -1.13995 | 1.40224 | 0.011845 |
| 1.62_142.0658m/z | HMDB0003447 | Tryptophanol | -2.43431 | 1.9939 | 3.19E-05 |
| 1.63_116.0501m/z | HMDB0000738 | Indole | -2.07243 | 2.0612 | 7.84E-05 |
| 1.63_170.0839n | HMDB0029747 | 2-Amino-5-phenylpyridine | -1.72835 | 2.31667 | 6.36E-06 |
| 1.63_171.0922m/z | HMDB0030533 | Alline | -1.63795 | 2.29023 | 8.81E-06 |
| 1.63_242.0928m/z | HMDB0029083 | Tryptophyl-Glycine | -2.39705 | 2.22807 | 6.81E-06 |
| 1.63_327.0707m/z | HMDB0128008 | 6-(5-ethenyl-2,3-dihydroxyphenoxy)-3,4,5-trihydroxyoxane-2-carboxylic acid | -2.10205 | 2.32189 | 4.54E-06 |
| 1.70_463.1840n | HMDB0060820 | Dihydroisomorphine-3-glucuronide | 2.224455 | 1.22151 | 0.017058 |
| 1.73_132.0814m/z | HMDB0001387 | N-Methylphenylethanolamine | -1.44317 | 2.2395 | 2.02E-05 |
| 1.73_146.0610m/z | HMDB0005785 | Indole-3-carbinol | -1.24396 | 1.8572 | 0.000507 |
| 1.73_176.0712m/z | HMDB0000466 | 3-Methylindole | -1.76168 | 2.21352 | 1.86E-05 |
| 1.73_203.0821m/z | HMDB0028848 | Glycyl-Phenylalanine | -1.39423 | 2.18723 | 3.88E-05 |
| 1.73_271.0696m/z | HMDB0000157 | Hypoxanthine | -1.35715 | 2.24253 | 1.58E-05 |
| 1.73_323.0320m/z | HMDB0038283 | 6-Chlorocatechin | -2.15464 | 2.21964 | 7.25E-05 |
| 1.73_339.0571m/z | HMDB0130026 | 3,4,5-trihydroxy-6-[(3-methoxy-3-oxopropanoyl)oxy]oxane-2-carboxylic acid | -2.17729 | 2.29684 | 1.28E-06 |
| 1.73_407.0446m/z | HMDB0129590 | [4-(5-hydroxy-7-methoxy-8-methyl-4-oxo-4H-chromen-3-yl)-2-methoxyphenyl]oxidanesulfonic acid | -2.18992 | 2.36685 | 9.04E-07 |
| 1.73_429.1539m/z | HMDB0012245 | Kinetin | -5.36403 | 1.70734 | 0.002013 |
| 1.73_475.0321m/z | HMDB0127328 | {2-[5,7-dihydroxy-2-(3-hydroxyphenyl)-4-oxo-4H-chromen-6-yl]-3-hydroxy-6-methyl-5-oxooxan-4-yl}oxidanesulfonic acid | -2.1227 | 2.15628 | 8.63E-06 |
| 1.76_333.0394m/z | HMDB0030756 | 13alpha-Hydroxydolineone | -1.01604 | 1.10966 | 0.029038 |
| 1.78_449.2346m/z | HMDB0014565 | Spironolactone | 1.595862 | 1.5752 | 0.004665 |
| 1.80_354.1190n | HMDB0031758 | Allithiamine | 1.284896 | 1.64601 | 0.00196 |
| 1.81_601.2454m/z | HMDB0005765 | Ophthalmic acid | 1.362159 | 1.22108 | 0.026344 |
| 1.91_331.2089m/z | HMDB0037162 | 1,2,3-Tris(1-ethoxyethoxy)propane | -1.4102 | 1.45014 | 0.004673 |
| 1.94_600.2651m/z | HMDB0035909 | Glaucarubol 15-O-beta-D-glucopyranoside | -1.47169 | 1.86364 | 0.002936 |
| 1.95_329.1932m/z | HMDB0028710 | Arginyl-Hydroxyproline | -1.42431 | 1.24504 | 0.012942 |
| 1.98_169.0112m/z | HMDB0000208 | Oxoglutaric acid | -4.50032 | 1.37102 | 0.008443 |
| 10.07_768.5529m/z | HMDB0007947 | PC(15:0/20:3(5Z,8Z,11Z)) | 1.769435 | 1.26869 | 0.011473 |
| 10.07_814.4998m/z | HMDB0012395 | PS(18:1(9Z)/22:6(4Z,7Z,10Z,13Z,16Z,19Z)) | 1.028754 | 1.37903 | 0.040135 |
| 10.31_778.5596m/z | HMDB0000564 | PC(16:0/16:0) | 1.034242 | 1.20988 | 0.008694 |
| 10.94_266.1501m/z | HMDB0015299 | Procarbazine | 2.251604 | 1.12015 | 0.023877 |
| 11.90_968.8596m/z | HMDB0043116 | TG(15:0/22:0/20:3(5Z,8Z,11Z)) | 1.928558 | 1.46867 | 0.008025 |
| 2.08_255.1134m/z | HMDB0015656 | Naphazoline | 1.061775 | 1.7437 | 0.000188 |
| 2.33_561.2886m/z | HMDB0038546 | ent-6R,16bOH,17-Trihydroxy-7-oxo-6,7-seco-19,6-kauranolide 6-O-glucoside | -1.63658 | 1.96859 | 0.000595 |
| 2.50_231.0326m/z | HMDB0127988 | (4-ethyl-2-methoxyphenyl)oxidanesulfonic acid | -5.36409 | 1.25294 | 0.014149 |
| 2.60_437.1614m/z | HMDB0030655 | Calabaxanthone | 1.15157 | 1.16894 | 0.018163 |
| 2.65_347.1596m/z | HMDB0137042 | 3-(4-hydroxy-3-methoxyphenyl)-N-[2-(4-hydroxyphenyl)ethyl]oxirane-2-carboximidic acid | 2.590896 | 1.62047 | 0.003841 |
| 2.66_369.1419m/z | HMDB0014815 | Isoflurophate | 1.86848 | 1.05767 | 0.046549 |
| 2.66_537.3045m/z | HMDB0029618 | Macrophorin D | 1.753105 | 2.0597 | 0.000156 |
| 2.67_547.9490m/z | HMDB0060713 | 2-Chloro-2'-deoxyadenosine-5'-triphosphate | 1.045772 | 1.19332 | 0.028528 |
| 2.69_563.2921m/z | HMDB0030312 | Floribundine | 1.757564 | 1.44543 | 0.007217 |
| 2.71_577.2989m/z | HMDB0060623 | Perindopril Acyl-beta-D-glucuronide | 1.849728 | 1.7588 | 0.00083 |
| 2.83_225.1128m/z | HMDB0035240 | Dihydroactinidiolide | 2.857623 | 1.13891 | 0.03025 |
| 2.84_183.0931m/z | HMDB0029834 | Harmalan | -3.47999 | 1.40909 | 0.005118 |
| 2.84_223.0287m/z | HMDB0031162 | 3,3'-Thiobispropanoic acid | 5.559146 | 1.15687 | 0.03941 |
| 2.86_369.1412m/z | HMDB0014815 | Isoflurophate | 2.722029 | 1.73652 | 0.001207 |
| 2.88_347.1592m/z | HMDB0031929 | Zanthodioline | 1.253616 | 1.32449 | 0.031911 |
| 2.92_223.0275m/z | HMDB0031162 | 3,3'-Thiobispropanoic acid | 1.642513 | 1.40894 | 0.018075 |
| 3.04_481.1853m/z | HMDB0034463 | Austin | 1.04558 | 1.13396 | 0.035021 |
| 3.06_299.1386m/z | HMDB0014643 | Tolmetin | 1.760254 | 1.44219 | 0.018509 |
| 3.07_547.2348m/z | HMDB0014433 | Amcinonide | 1.667442 | 1.11675 | 0.034759 |
| 3.10_429.1910m/z | HMDB0006224 | 17-beta-Estradiol-3-glucuronide | 1.811373 | 1.41996 | 0.025571 |
| 3.12_447.2892m/z | HMDB0062305 | 1-(11Z-eicosenoyl)-glycero-3-phosphate | -1.38081 | 1.33045 | 0.010336 |
| 3.14_351.0542m/z | HMDB0135218 | (3-oxo-1,3-diphenylpropoxy)sulfonic acid | 2.233762 | 1.12601 | 0.026114 |
| 3.15_277.1870m/z | HMDB0028952 | Lysyl-Hydroxyproline | -1.42837 | 1.66329 | 0.004635 |
| 3.17_215.1288m/z | HMDB0035837 | Citronellic acid | -1.72532 | 1.34422 | 0.04223 |
| 3.22_253.1339m/z | HMDB0033428 | (+)-Setoclavine | 1.182992 | 1.4102 | 0.010509 |
| 3.26_354.1651n | HMDB0015548 | Valganciclovir | -1.39097 | 1.07254 | 0.03352 |
| 3.29_272.1281m/z | HMDB0041836 | Benzoyl ecgonine | 3.226328 | 1.13005 | 0.039264 |
| 3.29_341.0675m/z | HMDB0034126 | Gyrocyanin | 3.660615 | 1.12113 | 0.045925 |
| 3.29_583.2560m/z | HMDB0037774 | Coriandrone A | 4.493937 | 1.14043 | 0.029097 |
| 3.31_292.0747n | HMDB0039404 | 3-Hydroxy-9-(4-hydroxyphenyl)-1H,3H-naphtho[1,8-cd]pyran-1-one | -1.49967 | 1.56232 | 0.008786 |
| 3.33_253.1344m/z | HMDB0033428 | (+)-Setoclavine | 1.002177 | 1.32238 | 0.012031 |
| 3.36_505.3306m/z | HMDB0114773 | PA(10:0/13:0) | -1.43479 | 1.45434 | 0.005324 |
| 3.42_163.1473m/z | HMDB0031181 | Homodihydrojasmone | 1.996888 | 1.24285 | 0.033339 |
| 3.42_291.0693m/z | HMDB0135230 | (1,3-diphenylpropoxy)sulfonic acid | -1.84379 | 1.71613 | 0.001427 |
| 3.42_359.0564m/z | HMDB0060627 | Malaoxon | -1.06283 | 1.10792 | 0.028254 |
| 3.44_157.0868m/z | HMDB0000451 | cis-4-Hydroxycyclohexylacetic acid | 2.346544 | 1.1626 | 0.045615 |
| 3.53_316.1330n | HMDB0060803 | 8-Hydroxydesmethylclomipramine | 1.518966 | 1.58148 | 0.001521 |
| 3.53_511.2905m/z | HMDB0060527 | Kukoamine D | 1.014029 | 1.31946 | 0.004728 |
| 3.54_653.2224m/z | HMDB0014552 | Loxapine | 2.158184 | 1.52186 | 0.004718 |
| 3.55_302.3047m/z | HMDB0059835 | Nonanal | -3.76882 | 1.23317 | 0.038791 |
| 3.68_446.2909m/z | HMDB0000138 | Glycocholic acid | -1.62258 | 1.21219 | 0.024948 |
| 3.68_759.0294m/z | HMDB0125287 | [3-(3,5,7-trihydroxy-4-oxo-3,4-dihydro-2H-1-benzopyran-2-yl)phenyl]oxidanesulfonic acid | 1.4343 | 1.31521 | 0.022429 |
| 3.70_499.1587m/z | HMDB0035664 | Dukunolide B | 1.20908 | 1.30937 | 0.03036 |
| 3.72_297.0463m/z | HMDB0124938 | 6-(carboxymethoxy)-3,4,5-trihydroxyoxane-2-carboxylic acid | 1.32277 | 2.05813 | 5.97E-05 |
| 3.75_272.1107m/z | HMDB0060841 | N-Despropyl-rotigotine | 1.197192 | 1.37862 | 0.022638 |
| 3.83_621.4135m/z | HMDB0116636 | PG(a-13:0/a-13:0) | -1.49564 | 1.44787 | 0.005716 |
| 3.85_211.0355m/z | HMDB0062567 | N-phosphocreatinate(2-) | 1.042013 | 1.42582 | 0.01882 |
| 3.87_700.6597m/z | HMDB0004955 | Cer(d18:1/26:0) | 1.118662 | 1.19439 | 0.036722 |
| 3.90_517.2315m/z | HMDB0038923 | Cinnamoside | -3.28301 | 1.09552 | 0.038324 |
| 3.91_415.3428m/z | HMDB0011544 | MG(0:0/20:2(11Z,14Z)/0:0) | -1.18066 | 1.91143 | 0.000534 |
| 4.05_449.1735m/z | HMDB0035062 | Arnamiol | -1.2609 | 1.34705 | 0.009339 |
| 4.06_269.1537m/z | HMDB0029639 | (4-Methylphenyl)acetaldehyde | 3.125477 | 1.25811 | 0.024199 |
| 4.07_393.2275m/z | HMDB0029422 | L-Histidine trimethylbetaine | -1.14859 | 1.09264 | 0.037916 |
| 4.14_227.0920m/z | HMDB0038181 | Peperinic acid | -1.38543 | 1.62278 | 0.002473 |
| 4.15_301.1651m/z | HMDB0031094 | Glycerol tributanoate | -1.00705 | 1.26891 | 0.023595 |
| 4.20_159.0807m/z | HMDB0002043 | 5-Phenylvaleric acid | -1.8406 | 1.46885 | 0.011338 |
| 4.20_277.1438m/z | HMDB0035906 | Alantolactone | -1.56901 | 1.3862 | 0.037797 |
| 4.29_241.1072m/z | HMDB0036226 | 2,6-Dimethoxy-4-propylphenol | -1.18476 | 1.3521 | 0.022473 |
| 4.33_380.2423m/z | HMDB0030026 | Isohumulone A | -2.29655 | 1.44232 | 0.006888 |
| 4.33_737.4959m/z | HMDB0116637 | PG(a-13:0/18:2(9Z,11Z)) | -1.80109 | 1.34122 | 0.011371 |
| 4.47_547.1984m/z | HMDB0240205 | 锘緽osutinib | -1.25939 | 1.20146 | 0.028196 |
| 4.49_99.0297m/z | HMDB0029616 | Diazenedicarboxamide | 1.24649 | 2.01004 | 0.00034 |
| 4.56_297.1130m/z | HMDB0035018 | 2-Phenylethyl 3-phenyl-2-propenoate | -1.708 | 1.43302 | 0.035051 |
| 4.57_175.0760m/z | HMDB0041666 | 3-Hydroxyphenyl-valeric acid | -1.4684 | 1.29544 | 0.008851 |
| 4.57_365.1002m/z | HMDB0000017 | 4-Pyridoxic acid | -2.71166 | 1.92546 | 0.027184 |
| 4.72_609.2957m/z | HMDB0005035 | Ondansetron | 1.34363 | 1.37629 | 0.0166 |
| 4.80_438.2834m/z | HMDB0037735 | Isoamyl 2-furonpropionate | -1.58392 | 1.47101 | 0.005272 |
| 4.88_409.2794m/z | HMDB0039974 | (1S,2S,4R,8R)-p-Menthane-1,2,8,9-tetrol | -1.08756 | 2.12654 | 1.56E-05 |
| 4.89_268.2625m/z | HMDB0031078 | Pentadecanal | -1.50654 | 2.61648 | 1.82E-07 |
| 4.93_669.4155m/z | HMDB0038223 | Carnocin U I49 | -2.54133 | 1.35538 | 0.005293 |
| 5.02_301.2167m/z | HMDB0002190 | 5,6-Epoxy-8,11,14-eicosatrienoic acid | 3.16898 | 1.32315 | 0.007922 |
| 5.03_581.3613m/z | HMDB0014857 | Azatadine | -2.06537 | 1.66196 | 0.003507 |
| 5.06_562.3147m/z | HMDB0010387 | LysoPC(18:3(6Z,9Z,12Z)) | -1.13575 | 1.2952 | 0.024919 |
| 5.08_512.2993m/z | HMDB0010379 | LysoPC(14:0/0:0) | -1.51964 | 2.122 | 7.95E-05 |
| 5.08_559.2733m/z | HMDB0037844 | N-(1-Deoxy-1-fructosyl)valine | -1.08836 | 1.86364 | 0.000231 |
| 5.09_452.2778m/z | HMDB0011473 | LysoPE(0:0/16:0) | -1.32535 | 1.936 | 0.000486 |
| 5.13_183.0117m/z | HMDB0015435 | Polystyrene sulfonate | 1.745766 | 1.16805 | 0.040978 |
| 5.14_355.1110m/z | HMDB0015264 | Pioglitazone | 1.526035 | 1.60075 | 0.004364 |
| 5.15_382.1296m/z | HMDB0029332 | 5-Methoxynoracronycine | 1.613075 | 1.63303 | 0.003188 |
| 5.18_295.2274m/z | HMDB0031336 | (Z)-9-Cycloheptadecen-1-one | 1.480531 | 1.18818 | 0.007471 |
| 5.19_315.2535m/z | HMDB0000798 | Oenanthic ether | 1.058632 | 1.51892 | 0.010861 |
| 5.19_449.2845m/z | HMDB0011678 | Geranylgeranylcysteine | -1.98194 | 1.4192 | 0.011121 |
| 5.24_562.3150m/z | HMDB0010388 | LysoPC(18:3(9Z,12Z,15Z)) | -1.03479 | 1.594 | 0.005637 |
| 5.27_431.2421m/z | HMDB0029656 | Erinacine E | -1.30967 | 1.86139 | 0.000232 |
| 5.29_431.1896m/z | HMDB0036131 | S-Furanopetasitin | -1.89356 | 2.26004 | 2.57E-06 |
| 5.29_452.2782m/z | HMDB0011503 | LysoPE(16:0/0:0) | -1.24299 | 2.11226 | 9.24E-05 |
| 5.29_493.1196m/z | HMDB0135750 | [3-(4-methoxyphenyl)propoxy]sulfonic acid | 1.690591 | 1.5249 | 0.003528 |
| 5.29_512.2995m/z | HMDB0061691 | 1-Heptadecanoylglycerophosphoethanolamine | -1.39714 | 2.17403 | 0.000101 |
| 5.29_529.2878m/z | HMDB0014257 | E-10-Hydroxydesmethylnortriptyline | -1.43721 | 1.81515 | 0.000767 |
| 5.29_716.2615m/z | HMDB0012177 | 5-Methyltetrahydropteroyltri-L-glutamate | -1.80415 | 1.81426 | 0.000521 |
| 5.34_562.3148m/z | HMDB0010388 | LysoPC(18:3(9Z,12Z,15Z)) | -1.27794 | 1.46981 | 0.011863 |
| 5.44_455.2383m/z | HMDB0040390 | Erinacine B | 2.32127 | 1.23051 | 0.037034 |
| 5.51_655.3789m/z | HMDB0039892 | Cloversaponin I | -1.43771 | 1.22223 | 0.019965 |
| 5.52_195.9710m/z | HMDB0015373 | Chloroxine | 2.380549 | 1.2558 | 0.031177 |
| 5.53_538.3154m/z | HMDB0010383 | LysoPC(16:1(9Z)/0:0) | -1.09117 | 2.14044 | 0.000293 |
| 5.59_527.3160m/z | HMDB0015620 | Mianserin | -1.76436 | 1.50791 | 0.021119 |
| 5.61_1058.6352m/z | HMDB0116810 | CL(8:0/8:0/8:0/16:0) | -1.40354 | 1.3744 | 0.014636 |
| 5.61_1130.1316m/z | HMDB0034097 | Bicornin | -1.37245 | 1.43123 | 0.009676 |
| 5.63_572.2976m/z | HMDB0011494 | LysoPE(0:0/22:5(4Z,7Z,10Z,13Z,16Z)) | -1.80045 | 1.51775 | 0.017094 |
| 5.67_396.1455m/z | HMDB0030435 | Simulansamide | 1.088408 | 1.50154 | 0.005369 |
| 5.68_149.0088m/z | HMDB0001352 | Hydroxypyruvic acid | 1.116812 | 1.11739 | 0.019487 |
| 5.75_854.4988m/z | HMDB0009474 | PE(20:5(5Z,8Z,11Z,14Z,17Z)/22:6(4Z,7Z,10Z,13Z,16Z,19Z)) | 3.051717 | 1.20269 | 0.01742 |
| 5.81_472.2743m/z | HMDB0002200 | Leukotriene E4 | -2.38847 | 1.6461 | 0.001588 |
| 5.83_563.2883m/z | HMDB0033375 | Octyl gallate | -2.74986 | 1.95835 | 0.000515 |
| 5.85_421.2257m/z | HMDB0032386 | 2-Methylacetophenone | -2.24406 | 1.97971 | 0.000124 |
| 5.86_515.2882m/z | HMDB0029340 | Ceanothine C | -3.38838 | 1.98799 | 0.000443 |
| 5.90_1081.6327m/z | HMDB0116818 | CL(8:0/8:0/8:0/19:0) | -1.39325 | 1.45626 | 0.01737 |
| 5.96_539.2885m/z | HMDB0034617 | (3b,4b,11b,14b)-11-Ethoxy-3,4-epoxy-14-hydroxy-12-cyathen-15-al 14-xyloside | -2.57658 | 2.24011 | 4.19E-05 |
| 5.96_562.2616n | HMDB0036856 | 19-Hydroxycinnzeylanol 19-glucoside | -1.00999 | 1.45603 | 0.006223 |
| 6.03_492.3086m/z | HMDB0010383 | LysoPC(16:1(9Z)/0:0) | -1.0349 | 1.39696 | 0.017629 |
| 6.03_552.3298m/z | HMDB0011482 | LysoPE(0:0/20:1(11Z)) | -1.19972 | 1.77908 | 0.002247 |
| 6.04_515.2885m/z | HMDB0029340 | Ceanothine C | -3.06193 | 2.05962 | 0.000273 |
| 6.04_583.2758m/z | HMDB0031448 | Glycosides | -3.35614 | 1.56592 | 0.005532 |
| 6.07_526.2930m/z | HMDB0011525 | LysoPE(22:5(7Z,10Z,13Z,16Z,19Z)/0:0) | -1.06197 | 1.91424 | 0.005394 |
| 6.15_183.0112m/z | HMDB0015435 | Polystyrene sulfonate | 1.793792 | 1.28295 | 0.026095 |
| 6.16_463.2250m/z | HMDB0114755 | LysoPA(22:6(4Z,7Z,10Z,13Z,16Z,19Z)/0:0) | -1.60957 | 1.32617 | 0.03029 |
| 6.17_410.1610m/z | HMDB0031754 | Nequinate | 1.040208 | 1.44626 | 0.008089 |
| 6.28_820.5149m/z | HMDB0113377 | PE-NMe(20:3(5Z,8Z,11Z)/18:4(6Z,9Z,12Z,15Z)) | 1.335395 | 1.40818 | 0.017212 |
| 6.31_1066.1736m/z | HMDB0037822 | Tridodecylamine | -3.29707 | 1.4897 | 0.007034 |
| 6.31_1133.6613m/z | HMDB0116977 | CL(8:0/8:0/13:0/18:2(9Z,11Z)) | -1.5924 | 1.54152 | 0.004027 |
| 6.31_805.5037m/z | HMDB0030407 | Merodesmosine | -1.21427 | 1.46132 | 0.006343 |
| 6.37_554.2621m/z | HMDB0001045 | Enkephalin L | -3.2674 | 1.0652 | 0.038059 |
| 6.42_590.3443m/z | HMDB0010394 | LysoPC(20:3(8Z,11Z,14Z)) | -1.01749 | 1.44621 | 0.012031 |
| 6.49_405.1914m/z | HMDB0000037 | Aldosterone | -1.04829 | 1.48935 | 0.00577 |
| 6.55_491.2879m/z | HMDB0000413 | 3-Hydroxydodecanedioic acid | -2.78932 | 2.14648 | 0.000104 |
| 6.56_464.2770m/z | HMDB0010380 | LysoPC(14:1(9Z)) | -2.19408 | 1.67305 | 0.001061 |
| 6.57_351.2478m/z | HMDB0062762 | L-argininium(1+) | -1.08668 | 1.25428 | 0.012267 |
| 6.67_183.0112m/z | HMDB0062551 | 4-ethylphenylsulfate | 1.726886 | 1.42435 | 0.026356 |
| 6.69_449.2176m/z | HMDB0041953 | Nnal-N-oxide | -1.63517 | 2.08357 | 1.91E-05 |
| 6.74_517.3050m/z | HMDB0029829 | Norchalciporyl propionate | -2.83305 | 2.16413 | 8.06E-05 |
| 6.74_585.2978m/z | HMDB0005035 | Ondansetron | -3.18532 | 1.5347 | 0.007862 |
| 6.81_295.2268m/z | HMDB0031336 | (Z)-9-Cycloheptadecen-1-one | 1.04196 | 1.58934 | 0.009622 |
| 6.89_550.3505m/z | HMDB0010408 | LysoPC(P-18:1(9Z)) | -1.02143 | 1.75727 | 0.003275 |
| 6.97_554.2634m/z | HMDB0001045 | Enkephalin L | -2.61461 | 1.39815 | 0.020552 |
| 7.16_377.1465m/z | HMDB0028808 | Glutaminyltryptophan | 1.233108 | 1.39478 | 0.012279 |
| 7.37_745.5038m/z | HMDB0037045 | 19'-Hexanoyloxymytiloxanthin | -1.37666 | 1.6985 | 0.000832 |
| 7.43_473.3630m/z | HMDB0034505 | Soyasapogenol A | -1.32194 | 1.41764 | 0.003915 |
| 7.61_833.4170m/z | HMDB0041332 | Balagyptin | -1.12932 | 1.68776 | 0.004546 |
| 7.75_931.5076m/z | HMDB0002829 | Androsterone glucuronide | 1.747118 | 1.45251 | 0.003396 |
| 7.78_313.1759m/z | HMDB0015317 | Pergolide | 1.194241 | 1.40705 | 0.008155 |
| 7.98_535.3008m/z | HMDB0028889 | Histidinyl-Leucine | 1.555298 | 1.5818 | 0.001933 |
| 8.09_732.3847m/z | HMDB0116536 | PGP(i-12:0/i-12:0) | 1.215438 | 1.86767 | 0.000897 |
| 8.09_925.6326m/z | HMDB0039086 | Hydroxysintaxanthin 5,6-epoxide | 1.43638 | 1.95036 | 0.000283 |
| 8.10_857.6483m/z | HMDB0002728 | Thyroxine sulfate | 1.27883 | 1.8738 | 0.000996 |
| 8.11_573.4043m/z | HMDB0002890 | 3-cis-Hydroxy-b,e-Caroten-3'-one | 3.873101 | 1.74103 | 0.001577 |
| 8.20_1003.6758m/z | HMDB0039090 | 7',8'-Dihydro-8'-hydroxyreticulataxanthin | 1.25942 | 1.53509 | 0.006678 |
| 8.25_355.1575m/z | HMDB0014697 | Hydroxyzine | 1.087598 | 1.61186 | 0.001702 |
| 8.25_399.1233m/z | HMDB0030108 | Cudraflavone A | 1.341427 | 1.31002 | 0.022587 |
| 8.35_563.5035m/z | HMDB0000207 | Oleic acid | 1.181973 | 1.32613 | 0.01055 |
| 8.49_953.5449m/z | HMDB0034603 | Hebevinoside XIII | -1.30158 | 1.2964 | 0.02038 |
| 8.53_911.5667m/z | HMDB0009819 | PI(18:0/22:5(4Z,7Z,10Z,13Z,16Z)) | -2.75093 | 2.19976 | 1.18E-05 |
| 8.67_279.2330m/z | HMDB0000673 | Linoleic acid | 1.311621 | 1.29233 | 0.006733 |
| 8.88_435.3469m/z | HMDB0000520 | 5a-Cholestane-3a,7a,12a,25-tetrol | 1.464434 | 1.13328 | 0.035277 |
| 9.00_742.4800m/z | HMDB0009683 | PE(22:6(4Z,7Z,10Z,13Z,16Z,19Z)/16:1(9Z)) | 2.38281 | 1.37521 | 0.021461 |
| 9.00_810.4668m/z | HMDB0015111 | Telithromycin | 1.107239 | 1.40451 | 0.019897 |
| 9.10_615.4224m/z | HMDB0036966 | 28-Glucopyranosyl-3-methyloleanolic acid | 1.833222 | 1.26934 | 0.04043 |
| 9.37_593.4497m/z | HMDB0013250 | Myristoylglycine | -1.56021 | 1.04107 | 0.038233 |
| 9.68_766.5381m/z | HMDB0009291 | PE(20:2(11Z,14Z)/18:2(9Z,12Z)) | 2.949157 | 1.16155 | 0.008607 |
| 9.70_504.3085m/z | HMDB0011483 | LysoPE(0:0/20:2(11Z,14Z)) | 1.243114 | 1.09686 | 0.02528 |
| 9.88_766.5371m/z | HMDB0114355 | PE-NMe2(20:4(8Z,11Z,14Z,17Z)/16:0) | 1.16749 | 1.26273 | 0.008152 |

**Supplementary Table 2.** KEGG pathway of differential metabolites between the RPL and control groups.

| KEGG metabolic pathway | Total | Hits | Raw p | Holm adjust | FDR | Impact |
| --- | --- | --- | --- | --- | --- | --- |
| Lysine degradation | 30 | 4 | 0.00413 | 0.3302 | 0.33018 | 0.03067 |
| Tryptophan metabolism | 41 | 4 | 0.01275 | 1 | 0.50983 | 0.39282 |
| Butanoate metabolism | 15 | 2 | 0.04426 | 1 | 0.99735 | 0 |
| Histidine metabolism | 16 | 2 | 0.04987 | 1 | 0.99735 | 0.22131 |
| Tyrosine metabolism | 42 | 3 | 0.06843 | 1 | 1 | 0.02502 |
| Linoleic acid metabolism | 5 | 1 | 0.10931 | 1 | 1 | 1 |
| Valine, leucine and isoleucine biosynthesis | 8 | 1 | 0.16923 | 1 | 1 | 0 |
| Vitamin B6 metabolism | 9 | 1 | 0.18831 | 1 | 1 | 0 |
| Biosynthesis of unsaturated fatty acids | 36 | 2 | 0.19782 | 1 | 1 | 0 |
| Glycerophospholipid metabolism | 36 | 2 | 0.19782 | 1 | 1 | 0.04289 |
| Biotin metabolism | 10 | 1 | 0.20697 | 1 | 1 | 0 |
| Purine metabolism | 70 | 3 | 0.21284 | 1 | 1 | 0.07957 |
| Arginine biosynthesis | 14 | 1 | 0.27753 | 1 | 1 | 0 |
| Nicotinate and nicotinamide metabolism | 15 | 1 | 0.29419 | 1 | 1 | 0.1943 |
| Steroid hormone biosynthesis | 87 | 3 | 0.31984 | 1 | 1 | 0.01022 |
| Fructose and mannose metabolism | 20 | 1 | 0.37205 | 1 | 1 | 0 |
| Ether lipid metabolism | 20 | 1 | 0.37205 | 1 | 1 | 0 |
| Citrate cycle (TCA cycle) | 20 | 1 | 0.37205 | 1 | 1 | 0.05856 |
| beta-Alanine metabolism | 21 | 1 | 0.38659 | 1 | 1 | 0 |
| Folate biosynthesis | 27 | 1 | 0.46718 | 1 | 1 | 0 |
| Lipoic acid metabolism | 28 | 1 | 0.47957 | 1 | 1 | 0 |
| Alanine, aspartate and glutamate metabolism | 28 | 1 | 0.47957 | 1 | 1 | 0.04808 |
| Sphingolipid metabolism | 32 | 1 | 0.5264 | 1 | 1 | 0.21576 |
| Glyoxylate and dicarboxylate metabolism | 32 | 1 | 0.5264 | 1 | 1 | 0.21958 |
| Cysteine and methionine metabolism | 33 | 1 | 0.53745 | 1 | 1 | 0 |
| Glycine, serine and threonine metabolism | 33 | 1 | 0.53745 | 1 | 1 | 0.04653 |
| Valine, leucine and isoleucine degradation | 40 | 1 | 0.60807 | 1 | 1 | 0 |
| Amino sugar and nucleotide sugar metabolism | 42 | 1 | 0.62625 | 1 | 1 | 0 |
| Arachidonic acid metabolism | 44 | 1 | 0.6436 | 1 | 1 | 0.01474 |
| Primary bile acid biosynthesis | 46 | 1 | 0.66017 | 1 | 1 | 0.00805 |
| Drug metabolism - cytochrome P450 | 55 | 1 | 0.72591 | 1 | 1 | 0 |
